# Myosin 15 participates in assembly and remodeling of the presynapse

**DOI:** 10.1083/jcb.202305059

**Published:** 2025-07-08

**Authors:** Astrid G. Petzoldt, Marc J. F. Escher, Oriane Turrel, Niclas Gimber, Ina M. Schedina, Sophie Walter, Torsten W.B. Götz, Marta Maglione, David Toppe, Tanja Matkovic-Rachid, Alexander Neumann, Janine Lützkendorf, Jan Schmoranzer, Martin Lehmann, Jörg Großhans, Stephan J. Sigrist

**Affiliations:** 1 Institute of Biology, Freie Universität Berlin, Berlin, Germany; 2 Advanced Medical Bioimaging Core Facility, Charité-Universitätsmedizin, Berlin, Germany; 3 Leibniz-Forschungsinstitut für Molekulare Pharmakologie im Forschungsverbund Berlin e.V. (FMP), Berlin, Germany; 4 Department of biology, Philipps-Universität Marburg, Marburg, Germany; 5 NeuroCure, Charité-Universitätsmedizin, Berlin, Germany

## Abstract

The assembly and remodeling of presynaptic specializations are of crucial importance for circuit development and adaptive behaviors. However, the mechanisms by which presynaptic material is locally distributed within synaptic terminals and across consuming active zones remain poorly understood. In this study, we identify the conserved unconventional class XV myosin, Myo15, an actin motor, as a novel regulator of presynaptic assembly and remodeling in *Drosophila*. Myo15 localizes to the local actin and microtubule network at synaptic terminals. Depletion of Myo15 resulted in smaller individual active zones, increased active zone density, and irregular terminal morphology, while its overexpression enlarged individual active zones and promoted synaptic terminal growth. Myo15 was found to modulate the actin meshwork, and deletion of its microtubule-binding MyTH4 domain rendered the protein nonfunctional. Furthermore, Myo15 was essential for presynaptic functional homeostatic plasticity and memory consolidation. These findings suggest that Myo15 plays a critical role in the assembly and remodeling of presynaptic active zones.

## Teaser

The authors identify the conserved unconventional class XV myosin, Myo15, an actin motor, as a novel regulator of presynaptic assembly and remodeling in *Drosophila*. They find Myo15 to be essential for presynaptic functional homeostatic plasticity and memory consolidation.

## Introduction

Strength and specificity of neuronal connections depend on size, number, and spatial distribution of synapses. The precise regulation of synapse formation and plastic remodeling is crucial for neuronal function. At the presynapse, the active zone (AZ) serves as a specialized proteinaceous nanodomain that orchestrates neurotransmitter release. Its formation, maintenance, and remodeling require a continuous supply of newly synthesized material, including AZ-scaffolding proteins, synaptic vesicle (SV) proteins, release factors, and voltage-gated calcium (Ca^2+^) channels, primarily produced in the soma and transported along microtubules (MTs) to the consuming synaptic terminals (Sudhof, 2012; Chia et al., 2013a; Maeder et al., 2014; Petzoldt et al., 2016). Concerted delivery and distribution of presynaptic cargo are crucial for synaptogenesis, and their disruption is linked to neurodevelopmental and neurodegenerative diseases (Salinas et al., 2008; Waites and Garner, 2011; Franker and Hoogenraad, 2013). Presynaptic components are thought to be preassembled into transport vesicles (TVs) before reaching consuming AZs (Ahmari et al., 2000; Fejtova and Gundelfinger, 2006, Chia et al., 2013a; Rizalar et al., 2021). Impaired TV transport, whether through loss of the anterogradely transporting kinesin KIF1α/UNC104, its adaptor Arl8, or interference with TV biogenesis, reduces synapse number, synaptic terminal size, and synaptic transmission (Hall and Hedgecock, 1991, Zhang et al., 2016; Vukoja et al., 2018; Gotz et al., 2021). Conversely, increased transport rates or enhanced JNK/Fos signaling can augment synapse number (Collins et al., 2006; Vukoja et al., 2018; Goel et al., 2019). At the synaptic terminal, presynaptic cargo undergoes local redistribution between cytoplasmic pools and consuming presynapses (Giagtzoglou et al., 2009; Graf et al., 2009), also driving plastic presynapse remodeling. While this remodeling is critical for synaptic plasticity, the molecular mechanisms controlling presynaptic cargo delivery, redistribution, and AZ incorporation remain poorly understood. Recent findings highlight the F-actin meshwork as a key regulator (Dillon and Goda, 2005; Kevenaar and Hoogenraad, 2015; Rust and Maritzen, 2015; Gentile et al., 2022), with the F-actin–binder spinophilin (NAB-1) recruiting “early” seeding factors (Chia et al., 2012) and formins such as DAAM-1 or Dia playing essential roles in synaptogenesis (Soykan et al., 2017; Szikora et al., 2017; Migh et al., 2018).

Myosins, a family of actin-based motors, are grouped into 20 classes with 40 genes described in humans (Berg et al., 2001; Foth et al., 2006; Odronitz and Kollmar, 2007; Li et al., 2016) and 14 conserved myosins in *Drosophila* assigned to 10 different classes (Yamashita et al., 2000; Tzolovsky et al., 2002; Sebe-Pedros et al., 2014). They play key roles in intracellular actin-based cargo trafficking, including endocytosis, exocytosis, the maintenance of actin-based structures, such as filopodia, force sensing, and protein localization during cellular processes, like left-right asymmetry establishment (Belyantseva et al., 2003; Les Erickson et al., 2003; O’Connell et al., 2007; McConnell and Tyska, 2010; Hammer and Sellers, 2011; Petzoldt et al., 2012; Tumbarello et al., 2013; Crawley et al., 2014; Juan et al., 2018). Myosins share a conserved head domain allowing actin binding and ATP hydrolyzation, a neck region containing IQ motifs (isoleucine-glutamine motifs) that regulate activity, and a highly diverse tail region (Sellers, 2000; Lu et al., 2014; Li et al., 2016; Fili and Toseland, 2019). Their functional specificity is determined through their unique tail regions mediating protein interactions, in example via SH3 (Src homology 3) and PDZ (PSD-95, Dlg, ZO-1) domains, lipid-binding, via PH (pleckstrin homology) domains (Tzolovsky et al., 2002; Li et al., 2016; Fili and Toseland, 2019), or MyTH4-FERM (myosin tail homology 4–4.1 and ezrin/radixin/moesin) tandem domains acting as a structural “supramodule.” The MyTH4 domain enables MT interactions, while the FERM domain mediates cargo binding (Weber et al., 2004; Hirano et al., 2011; Lu et al., 2014; Planelles-Herrero et al., 2016).

To identify molecular regulators of presynaptic assembly and remodeling, we hypothesized that unconventional myosins, as actin-based motor proteins, could play an important role during synaptogenesis. We identified the class XV myosin, Myo15, as a novel regulator of synapse assembly and remodeling by performing genetic manipulations of Myo15 and presynaptic homeostatic plasticity (PHP) treatments, followed by immunofluorescence and functional electrophysiological analysis of the presynapse at the *Drosophila* neuromuscular junction (NMJ). We demonstrate that Myo15 regulates AZ size and number at the NMJ, NMJ morphology, reorganizes the terminal actin meshwork, and functionally relies on its MyTH4 domains, allowing MT binding. Importantly, Myo15 was required for homeostatic plasticity in response to pharmacological blockade of postsynaptic glutamate receptors and was essential for adult memory consolidation. Thus, Myo15 emerges as an important molecular regulator of AZ assembly and remodeling.

## Results

### Class XV Myo15 controls AZ size and number as well as NMJ morphology

**Figure 1.**
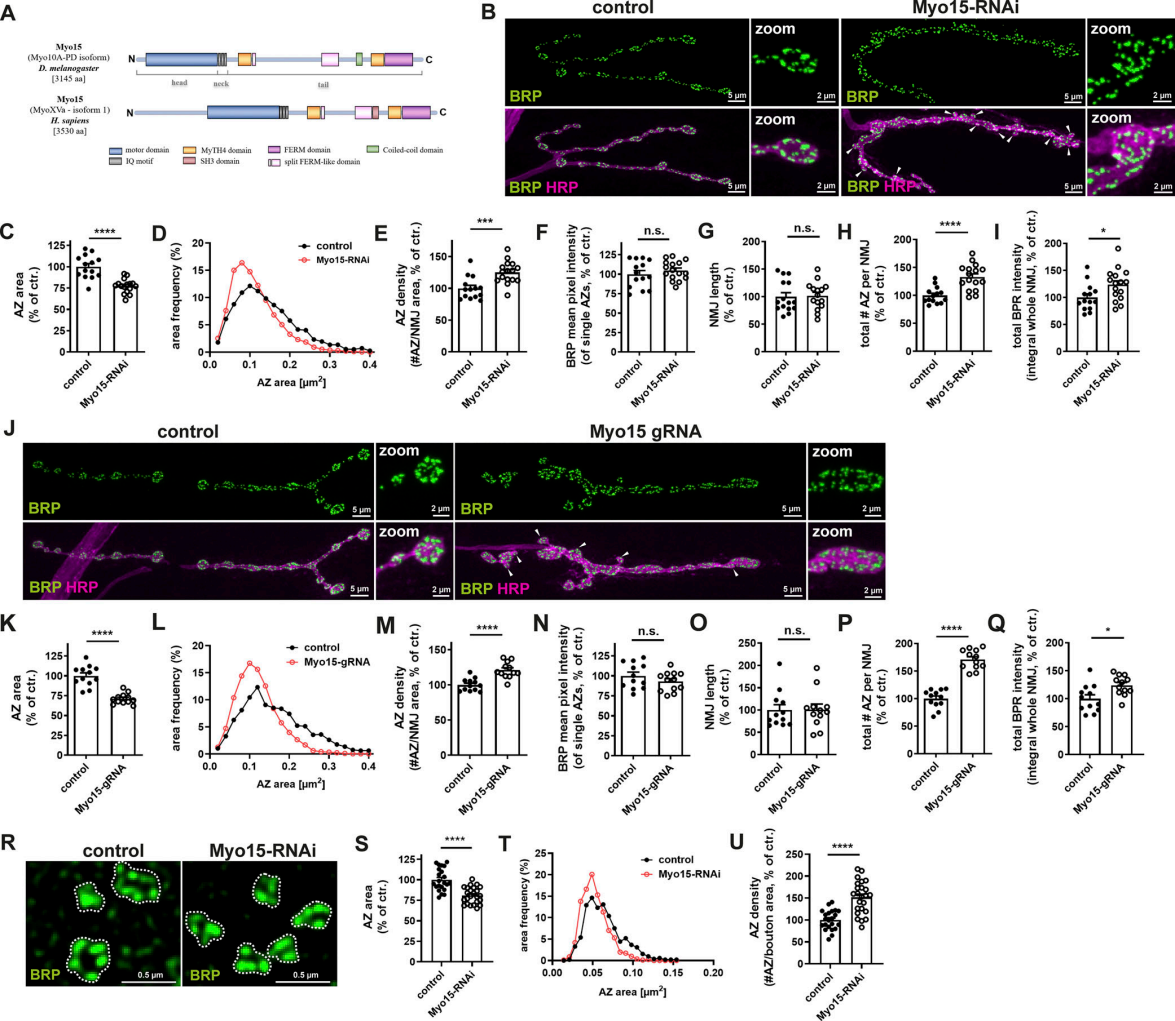
**Class XV Myo15 regulates NMJ morphology, presynaptic AZ scaffold size, and number. (A)** Schematic representation of class XV Myo15 in *D. melanogaster* and human domain structure. **(B)** Confocal images of RNAi-mediated Myo15-knock-down (UAS–Gal4 system using ok6-Gal4 driver) and control NMJs (driver control) stained for BRP (green) and HRP (magenta), scale bar overview 5 µm, zoom 2 µm, white arrowheads mark ectopic satellite boutons. **(C–I)** Quantifications of B. **(C)** AZ area (control 100.0 ± 3.63% *n* = 14; Myo15-RNAi 77.31 ± 2.06%, *n* = 16). **(D)** Relative frequency histogram of AZ areas (total number of AZs control 3034 and Myo15-RNAi 4738), bin width 0.02. **(E)** Density of AZs, as number of AZ per NMJ area (control 100.0 ± 4.77% *n* = 14; Myo15-RNAi 125.1 ± 4.38%, *n* = 16). **(F)** BRP mean pixel intensity of single AZs (control 100.0 ± 4.64% *n* = 14; Myo15-RNAi 104.8 ± 3.04%, *n* = 16). **(G)** NMJ length (control 100.0 ± 7.37% *n* = 15; Myo15-RNAi 101.6 ± 6.40%, *n* = 15). **(H)** Total number of all AZs per NMJ (control 100.0 ± 3.92% *n* = 14; Myo15-RNAi 132.9 ± 5.77%, *n* = 16). **(I)** Total BRP intensity as integral of the BRP mean pixel intensity over the entire NMJ (control 100.0 ± 6.68% *n* = 14; Myo15-RNAi 123.6 ± 7.27%, *n* = 16). **(J)** Confocal images of gRNA (CRISPR/Cas9)-mediated knock-down of Myo15 and control NMJs stained for BRP as AZ marker (green) and HRP outlining the synaptic terminal (magenta), scale bar overview 5 µm, zoom 2 µm, white arrowheads mark satellite boutons. **(K–Q)** Quantification of J. **(K)** AZ area (control 100.0 ± 3.75% *n* = 12; Myo15gRNA 71.09 ± 1.94%, *n* = 12). **(L)** Relative frequency histogram of AZ areas (total number of AZs: control 3026 and Myo15gRNA 5265), bin width 0.02. **(M)** Density of AZs, as number of AZ per NMJ area (control 100.0 ± 2.57%, *n* = 12; Myo15gRNA 121.1 ± 3.27%, *n* = 12). **(N)** BRP mean pixel intensity of single AZs (control 100.0 ± 4.59%, *n* = 12; Myo15gRNA 92.90 ± 3.52%, *n* = 12). **(O)** NMJ length (control 100.0 ± 11.95%, *n* = 12; Myo15gRNA 101.1 ± 12.25%, *n* = 12). **(P)** Total number of all AZs per NMJ (control 100.0 ± 4.81%, *n* = 12; Myo15gRNA 171.0 ± 5.11%, *n* = 12). **(Q)** Total BRP intensity as integral of the BRP mean pixel intensity over the entire NMJ (control 100.0 ± 7.02%, *n* = 12; Myo15gRNA 123.9 ± 5.11%, *n* = 12). **(R)** STED imaging of AZs labeled with BRP (green) upon RNAi-mediated Myo15-knock-down (scale bar 0.5 µm). **(S–U)** Quantification of R. **(S)** AZ area (control 100.0 ± 2.9%, *n* = 22; Myo15-RNAi 81.01 ± 2.04%, *n* = 24), (T) relative frequency histogram of AZ areas (total number of AZs: control 520 and Myo15-RNAi 810), bin width 0.007, (U) density of AZs, as number of AZ per NMJ area (control 100.0 ± 4.79%, *n* = 22; Myo15-RNAi 151.7 ± 7.57%, *n* = 24). All data are provided as mean ± SEM. **(C–I and K–Q)***n* represents NMJs with 1–2 NMJs/animal from five to six animals, R: *n* = image, with 1–2 boutons/image and two to three images/NMJ, 2 NMJs/animal, three to four animals in total. *P < 0.05; **P < 0.01; ***P < 0.001; ****P < 0.0001. SH3, Src homology 3.

To investigate the mechanisms underlying AZ assembly and remodeling, we performed a presynaptic knock-down screen of *Drosophila* unconventional myosins in larval motoneurons, assessing AZ size, number, and distribution in third instar larvae (Table 1). This screen identified Myo15 (also known as Myo10A due to its polytene chromosome localization or Sisyphus [Liu et al., 2008]) as a crucial molecular regulator of AZ remodeling. Myo15 is the sole class XV myosin in *Drosophila* (Yamashita et al., 2000; Tzolovsky et al., 2002). Its head and tail domains are highly conserved in humans and mice, with the head domain sharing 50% identity and 65% similarity, and the tail showing 33% identity. *Drosophila* Myo15 displays the canonical class XV myosin tail architecture, including a MyTH4 (myosin tail homology 4) domain close to the neck, a split FERM-like domain and a MyTH4-FERM tandem domain near the C terminus (Liang et al., 1999; Lu et al., 2014; Planelles-Herrero et al., 2016; Rich et al., 2021) (Fig. 1 A). Notably, Myo15 had been previously reported to affect NMJ morphology (Rich et al., 2021).

**Table 1.** List of all tested myosins via RNAi–mediated knock-down using the ok6-Gal4 driver

Myosin name	CG number	chr	Viablity (larva/pupa)	NMJ defects
Myo1C (Myo61F)	9155	III	Viable	No
Myo1D (Myo31F)	7438	II	Viable	No
Myo95E	5501	III	Viable	No
Zipper	15792	II	Pupal lethal	elongated NMJs
Mhc	17927	II	Viable	mild deformations
NinaC	5125	II	Viable	mild deformations
Didum	2146	II	Viable	No
Jaguar	5695	III	Viable	No
Crinkled	7595	II	Viable	No
Myo28B1	6976	II	Viable	No
Myo15 (myosin XV, Myo10A, Sisyphus)	2174	X	Viable	this study
Dachs (Myo29D)	10595	II	Viable	No

We tested 12 myosins of the 14 predicted *Drosophila* myosins (Sebe-Pedros et al., 2014).

Myo15 knock-down was performed using two independent approaches: RNAi targeting two distinct Myo15 mRNA sequences (Fig. 1, B–I) and somatic CRISPR/Cas9-mediated locus inactivation (Fig. 1, J–Q), both yielded similar results. Myo15 knock-down induced significant morphological changes at the NMJ, including irregular synaptic terminals, unevenly shaped boutons, additional satellite boutons, and thickened inter-bouton regions (Fig. 1, B and J arrowheads). At the single AZ level, immunostaining for Bruchpilot (BRP), a core AZ component (Kittel et al., 2006b; Wagh et al., 2006) revealed a ∼25% reduction of the average area of AZs (Fig. 1, C, D, K, and L), while AZ density (AZs per NMJ area) increased by ∼20–25% (Fig. 1, E and M). However, BRP pixel intensities at individual AZs remained unchanged (Fig. 1, F and N). Despite unaltered synaptic terminal length (Fig. 1, G and O), the increase in AZ density led to higher total AZ numbers (Fig. 1, H and P), and an overall increase in BRP total protein levels across the NMJ (Fig. 1, I and Q). We next examined presynaptic nano-architecture upon Myo15 depletion using stimulated emission depletion (STED) microscopy (∼60-nm resolution). We observed a ∼25% reduction in AZ scaffold area (Fig. 1, R–T) and an increase in AZ density (Fig. 1 U), while the overall presynaptic scaffold architecture characterized by the typical C-terminal BRP ring (Kittel et al., 2006a; Fouquet et al., 2009) appeared unchanged.

**Figure S1.**
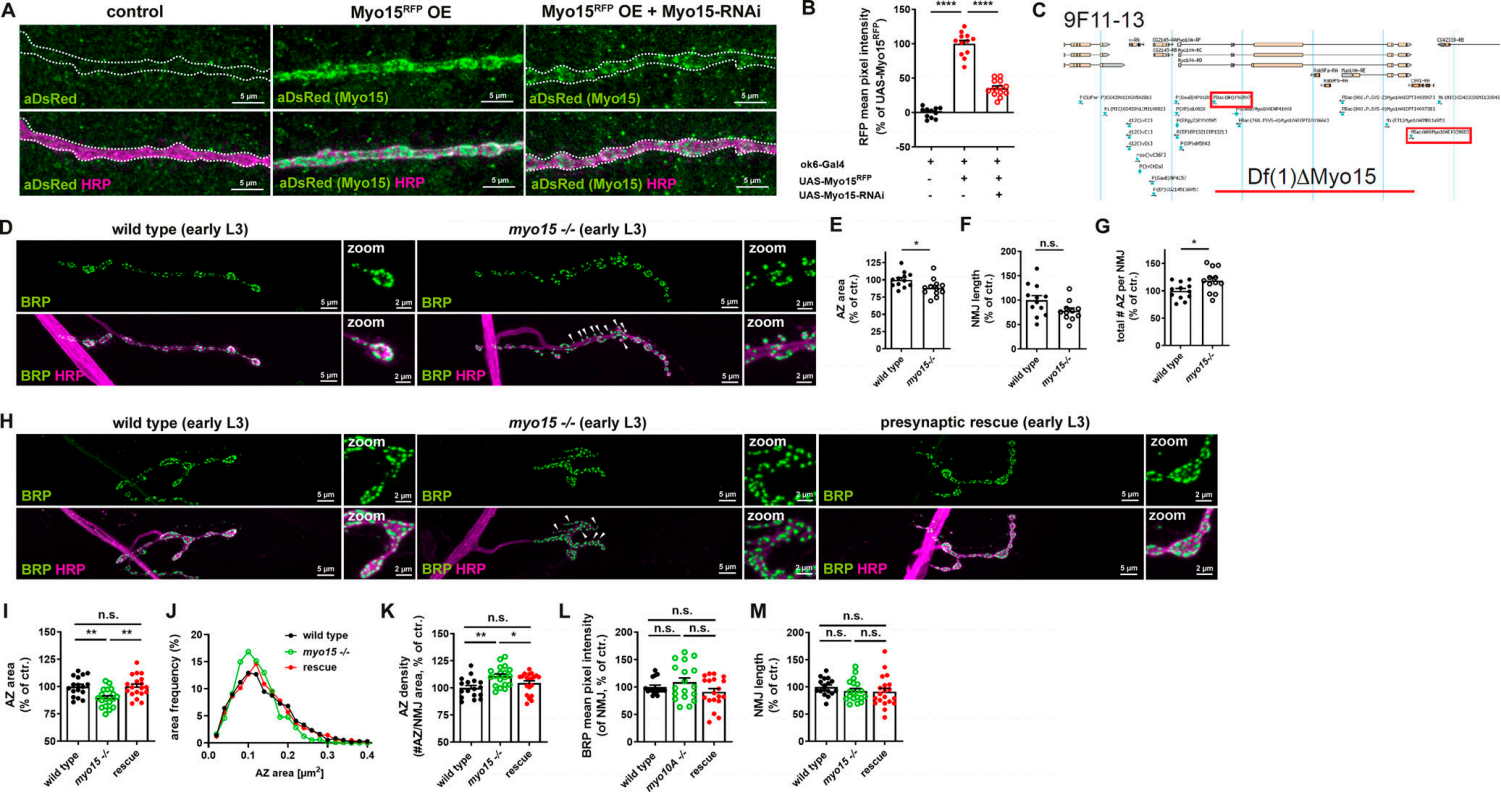
***Myo15*** ^**−/−**^ **mutants show defects in NMJ morphology and AZ scaffold assembly, which are rescued by presynaptic restoration of Myo15 level. (A)** Confocal images of overexpressed Myo15^RFP^ and simultaneous RNAi-mediated Myo15-knock-down show RNAi efficacy and specificity, stained for RFP with aDsRed (green) and HRP (magenta), scale bar overview 5 µm. **(B)** Quantifications of RFP mean pixel intensity of A (control 0.0 ± 2,26%, *n* = 11; Myo15^RFP^ (OE) 100 ± 4,98%, *n* = 12; Myo15^RFP^ (OE) and Myo15-RNAi 35,61 ± 3,21, *n* = 13). **(C)** Locus of Myo15 deletion (Df(1)ΔMyo15), red squares mark P-Elements used for flip-out (see Materials and methods for details, image adapted from https://flybase.org). **(D)** Confocal images of *myo15*^*−/−*^ mutant escapers and control NMJs stained for BRP (green) and HRP (magenta), scale bar overview 5 µm, zoom 2 µm, white arrowheads mark satellite boutons. **(E–G)** Quantifications of D. **(E)** AZ area (wild type 100.0 ± 3.20%, *n* = 12; *myo15*^*−/−*^ mutant 88.29 ± 3.61%, *n* = 12). **(F)** NMJ length (wild type 100.0 ± 9.31%, *n* = 12; *myo15*^*−/−*^ mutant 78.04 ± 6.04%, *n* = 11). **(G)** Total number of all AZs per NMJ (wild type 100.0 ± 4.45%, *n* = 12; *myo15*^*−/−*^ mutant 118.7 ± 6.34%, *n* = 12). **(H)** Confocal images of control, *myo15*^*−/−*^ mutant, and presynaptic rescue NMJs stained for BRP (green) and HRP (magenta), scale bar overview 5 µm, zoom 2 µm, white arrowheads mark satellite boutons. **(I–M)** Quantifications of H. **(I)** AZ area (wild type 100.0 ± 2.19%, *n* = 17; *myo15*^*−/−*^ mutant 89.70 ± 1.88%, *n* = 19; rescue 100.10 ± 2.26, *n* = 19). **(J)** Relative frequency histogram of AZ areas (total number of AZs control 1279, *myo15*^*−/−*^ mutant 1110, rescue 1237), bin width 0.02. **(K)** Density of AZs, as number of AZ per NMJ area (control 100.0 ± 2.25%, *n* = 17; *myo15*^*−/−*^ mutant 110.9 ± 2.08%, *n* = 19, rescue 104.40 ± 2.15, *n* = 20). **(L)** BRP mean pixel intensity of NMJ (control 100.0 ± 3.67%, *n* = 16; *myo15*^*−/−*^ mutant 108.80 ± 7.82%, *n* = 18, rescue 90.96 ± 6.29, *n* = 19). **(M)** NMJ length (control 100.0 ± 3.79%, *n* = 17; *myo15*^*−/−*^ mutant 92.76 ± 4.49%, *n* = 19, rescue 91.16 ± 6.34, *n* = 20). All data are provided as mean ± SEM. For all quantifications, n represents NMJs with 1–3 NMJs/animal from five to seven animals. *P < 0.05; **P < 0.01; ***P < 0.001; ****P < 0.0001. OE, overexpression.

To confirm specificity and efficacy of the RNAi-mediated Myo15 depletion, we overexpressed an RFP-tagged Myo15 construct while simultaneously inducing RNAi knock-down (Fig. S1 A). This resulted in a 60% reduction of Myo15-RFP signal (Fig. S1 B), validating the effectiveness of the RNAi approach.

We next attempted to verify the knock-down phenotype and generated a chromosomal *myo15*^*−/−*^ deletion mutant, using a flippase-mediated excision of the Myo15-coding region between two P-element insertions, followed by sequencing ([Thibault et al., 2004; Bellen et al., 2011] Fig. S1 C). Myo15 is essential for embryonic development in *Drosophila* due to its non-neuronal functions, particularly during dorsal closure (Liu et al., 2008). Maternal contribution of Myo15 confirmed by immunofluorescence staining of staged wild-type embryos (Liu et al., 2008) likely allows Myo15 null mutants to survive embryogenesis (Rich et al., 2021). Myo15 mRNA expression in larvae is highest in trachea (548 ± 80), hindgut (230 ± 19), and carcass (224 ± 21, https://flyatlas.org). Larval CNS expression is relatively low (34 ± 8). The homozygous *myo15*^*−/−*^ escaper larvae, generated here, displayed developmental delay, reduced size, and early L3-stage lethality, consistent with previous reports (Rich et al., 2021). These developmental defects likely derive from the non-neuronal Myo15 functions, and initial larval survival possibly results from maternal contribution. Despite developmental restraints, NMJ structure in escaper larvae resembled the knock-down phenotype, exhibiting irregularly shaped NMJs, satellite bouton formation, and thicker inter-bouton regions (Fig. S1 D, arrowheads). Like knock-down animals, mutants showed a decrease in AZ area (Fig. S1 E), unaltered NMJ length (Fig. S1 F), and an increase in total AZ numbers (Fig. S1 G).

To confirm that the observed defects were specific to Myo15 loss in motoneurons, we performed a rescue experiment by re-expressing full-length Myo15 in the motoneurons of *myo15*^*−/−*^ mutants using the motoneuron-specific driver *ok6-Gal4* (Fig. S1 H). Presynaptic rescue animals remained small and developmentally delayed, with a similarl early L3 lethality as the *myo15*^*−/−*^ mutants, likely due to the remaining loss of Myo15 in the non-neuronal tissue. However, presynaptic motoneuron-specific Myo15 re-expression restored NMJ morphology compared to *myo15*^*−/−*^ mutants, with regularly shaped NMJs without satellite boutons and smooth, oval-shaped boutons (Fig. S1 H). AZ size and density were restored to wild-type levels (Fig. S1, I–K), while BRP mean pixel intensity and NMJ length remained unchanged across all genetic conditions (Fig. S1, L and M).

### Loss of Myo15 affects AZ scaffold, SV protein accumulation, and early endosome structure

**Figure 2.**
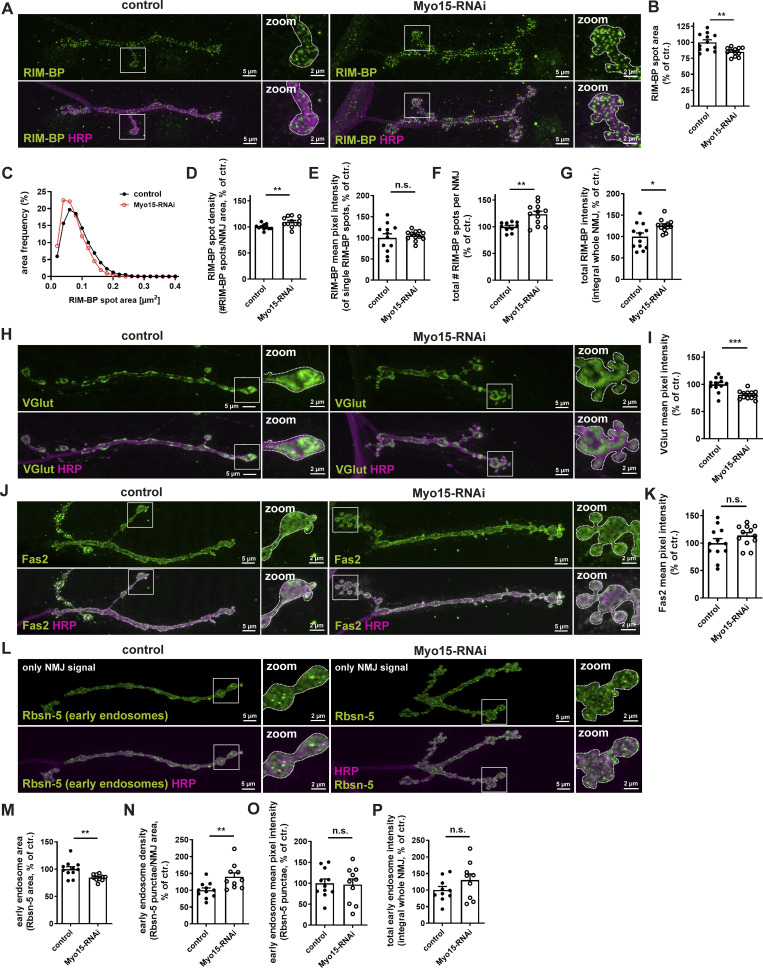
**Myo15 knock-down affects AZ scaffold, SVs, and EE. (A)** Confocal images of RNAi-mediated Myo15 knock-down and control NMJs stained for RIM-BP (green) and HRP (magenta). **(B–G)** Quantifications of A. **(B)** RIM-BP spot area (control 100.0 ± 3.88% *n* = 12; Myo15-RNAi 85.07 ± 1.92%, *n* = 12). **(C)** Relative frequency histogram of RIM-BP spot areas (total number of spots control 3610 and Myo15-RNAi 4428), bin width 0.02. **(D)** Density of RIM-BP spots, as number of spots per NMJ area (control 100.0 ± 1.66% *n* = 11; Myo15-RNAi 109.8 ± 2.75%, *n* = 12). **(E)** RIM-BP mean pixel intensity of single spots (control 100.0 ± 9.63% *n* = 12; Myo15-RNAi 105.3 ± 3.39%, *n* = 12). **(F)** Total number of RIM-BP spots per NMJ (control 100.0 ± 2.82% *n* = 10; Myo15-RNAi 123.4 ± 5.68%, *n* = 12). **(G)** Total RIM-BP intensity as integral of the RIM-BP mean pixel intensity over the entire NMJ (control 100.0 ± 8.52% *n* = 12; Myo15-RNAi 125,5 ± 4.23%, *n* = 12). **(H)** Confocal images of RNAi-mediated Myo15 knock-down and control NMJs stained for VGlut (green) and HRP (magenta). **(I)** Quantifications of H. VGlut mean pixel intensity of NMJ (control 100.0 ± 3.85% *n* = 12; Myo15-RNAi 80.87 ± 2.22%, *n* = 12). **(J)** Confocal images of RNAi-mediated Myo15 knock-down and control NMJs stained for Fas2 (green) and HRP (magenta). **(K)** Quantifications of J. Fas2 mean pixel intensity of NMJ (control 100.0 ± 8.23% *n* = 12; Myo15-RNAi 113.6 ± 5.40%, *n* = 12). **(L)** Confocal images of RNAi-mediated Myo15 knock-down and control NMJs stained for Rbsn-5 (green) and HRP (magenta), only showing the NMJ contribution. **(M–P)** Quantifications of L. **(M)** Rbsn-5 (early endosome [EE]) spot area (control 100.0 ± 4.51% *n* = 11; Myo15-RNAi 84.47 ± 1.87%, *n* = 10). **(N)** Density of Rbsn-5 spots, as number of spots per NMJ area (control 100.0 ± 6.97% *n* = 11; Myo15-RNAi 139.8 ± 11.81%, *n* = 10). **(O)** Rbsn-5 (EE) mean pixel intensity of single spots (control 100.0 ± 10.3% *n* = 11; Myo15-RNAi 96.68 ± 13.63%, *n* = 10). **(P)** Total Rbsn-5 (EE) intensity as integral of the Rbsn-5 mean pixel intensity over the entire NMJ (control 100.0 ± 10.84% *n* = 10; Myo15-RNAi 130.0 ± 18.67%, *n* = 9). For all images, scale bar overview 5 µm, zoom 2 µm. All data are provided as mean ± SEM. *N* represents NMJs with 1–2 NMJs/animal from five to six animals, *P < 0.05; **P < 0.01; ***P < 0.001; ****P < 0.0001. VGlut, vesicular glutamate transporter 1; Fas2, fasciclin 2.

**Figure S2.**
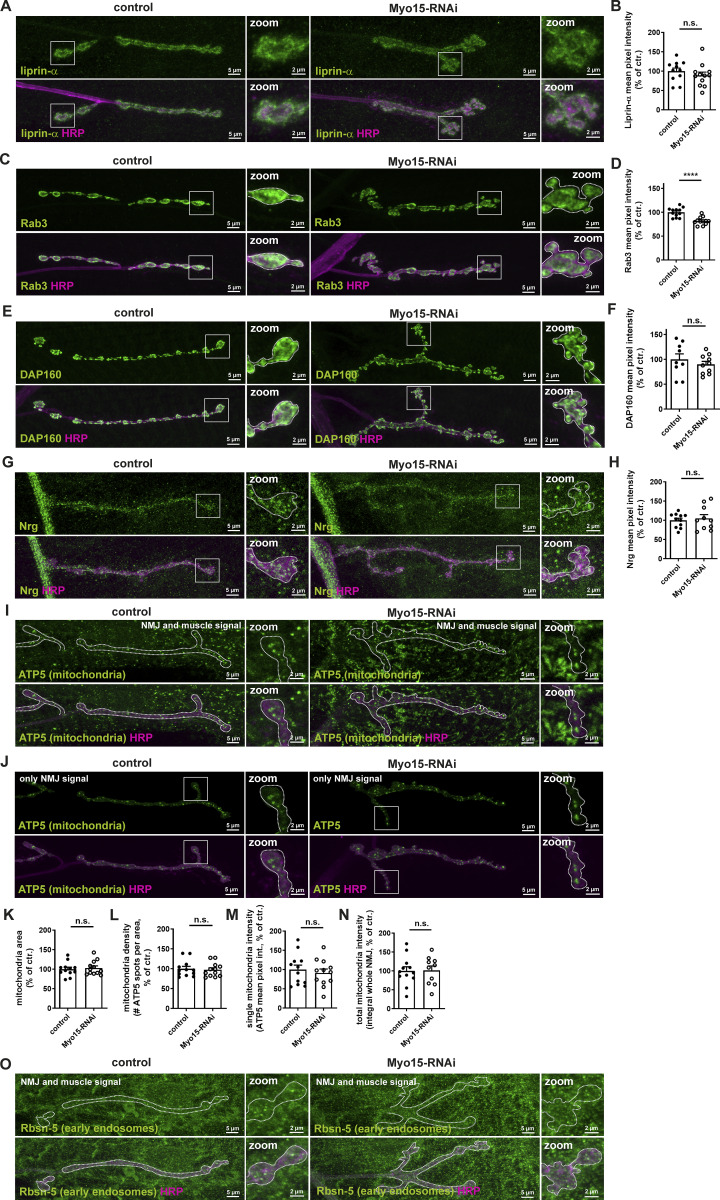
**Analysis of AZ, SV, endocytic and cell adhesion proteins, and mitochondria upon motoneuronal Myo15 knock-down. (A)** Confocal images of RNAi-mediated Myo15 knock-down and control NMJs stained for liprin-α (green) and HRP (magenta). **(B)** Quantifications of A. Liprin-α mean pixel intensity of NMJ (control 100.0 ± 8.04% *n* = 11; Myo15-RNAi 89.1 ± 8.38%, *n* = 12). **(C)** Confocal images of RNAi-mediated Myo15 knock-down and control NMJs stained for Rab3 (green) and HRP (magenta). **(D)** Quantifications of C. Rab3 mean pixel intensity of NMJ (control 100.0 ± 2.83% *n* = 12; Myo15-RNAi 82.0 ± 2.36%, *n* = 12). **(E)** Confocal images of RNAi-mediated Myo15 knock-down and control NMJs stained for DAP160 (green) and HRP (magenta). **(F)** Quantifications of E. DAP160 mean pixel intensity of NMJ (control 100.0 ± 11.0% *n* = 9; Myo15-RNAi 89.95 ± 6.04%, *n* = 10). **(G)** Confocal images of RNAi-mediated Myo15 knock-down and control NMJs stained for Nrg (green) and HRP (magenta). **(H)** Quantifications of G. Nrg mean pixel intensity of NMJ (control 100.0 ± 5.51% *n* = 11; Myo15-RNAi 105.0 ± 9.85%, *n* = 10). **(I and J)** Confocal images of RNAi-mediated Myo15 knock-down and control NMJs stained for ATP5 (mitochondria) (green) and HRP (magenta) with NMJ and muscle contribution, and (J) only NMJ ATP5 contribution. **(K–N)** Quantifications of J. **(K)** ATP5 spot (mitochondria) area (control 100.0 ± 5.13% *n* = 12; Myo15-RNAi 103.2 ± 5.22%, *n* = 12). **(L)** Density of ATP5 spots (mitochondria) as number of spots per NMJ area (control 100.0 ± 5.92% *n* = 12; Myo15-RNAi 97.35 ± 5.02%, *n* = 12). **(M)** ATP5 (mitochondria) mean pixel intensity of single spots (control 100.0 ± 11.67% *n* = 12; Myo15-RNAi 91.4 ± 10.06%, *n* = 12). **(N)** Total ATP5 (mitochondria) intensity as integral of the ATP5 mean pixel intensity over the entire NMJ (control 100.0 ± 11.04% *n* = 12; Myo15-RNAi 101.4 ± 11.71%, *n* = 10). **(O)** Confocal images of RNAi-mediated Myo15 knock-down and control NMJs stained for Rbsn-5 (EE) (green) and HRP (magenta) with NMJ and muscle contribution. For all images, scale bar overview 5 µm, zoom 2 µm. All data are provided as mean ± SEM. *N* represents NMJs with 1–2 NMJs/animal from five to six animals, *P < 0.05; **P < 0.01; ***P < 0.001; ****P < 0.0001. Nrg, neuroglian.

Having observed AZ scaffold changes in BRP immunostainings, we next examined the specificity of presynaptic biogenesis deficits in Myo15-deficient NMJ terminals. First, we analyzed RIM-binding protein (RIM-BP), a second conserved AZ scaffold protein that interacts with BRP to form concentric rings within the AZ scaffold, positioned above voltage-gated Ca^2+^ channels (Liu et al., 2011; Sudhof, 2012; Acuna et al., 2015; Tang et al., 2016; Petzoldt et al., 2020) (Fig. 2 A). Upon Myo15 knock-down, we observed that RIM-BP spot areas were significantly reduced (Fig. 2, B and C), spot density was increased (Fig. 2 D), and mean pixel intensity of RIM-BP spots remained unchanged (Fig. 2 E), mirroring the BRP phenotype. Given the increased AZ density and unchanged terminal size, the total number of AZs (RIM-BP spots, Fig. 2 F) and the total RIM-BP intensity across the NMJ (Fig. 2 G) were both increased, consistent with our BRP findings. While BRP accumulates rather late during presynaptic assembly, the evolutionary conserved liprin-α/SYD-2 protein plays a crucial role in early AZ assembly by recruiting several core AZ proteins (Fouquet et al., 2009; Owald et al., 2012; Sudhof, 2012; Chia et al., 2013b). Upon Myo15 knock-down, liprin-α protein levels at the NMJ remained unchanged (Fig. S2, A and B).

Next, we examined SV protein levels following Myo15 knock-down. We analyzed the vesicular glutamate transporter 1 (Santos et al., 2009) and the small GTPase Rab3, which regulates SV docking and exocytosis (Takamori et al., 2006; Binotti et al., 2016). Both SV markers showed a ∼20% reduction in protein levels at the NMJ (Fig. 2, H and I; and Fig. S2, C and D), suggesting that Myo15 may regulate SV quantity and/or distribution.

To address the endocytic molecular machinery orchestrating SV recycling in the proximity of AZs, we analyzed Dap160/intersectin (Pechstein et al., 2010) upon Myo15 knock-down but observed no changes in protein levels (Fig. S2, E and F). We next tested for cell adhesion molecules and found protein levels of both fasciclin 2 (Fas2), a conserved neural cell adhesion molecule (Grenningloh et al., 1991) (Fig. 2, J and K), and neuroglian (Nrg), a transsynaptic L1-type cell adhesion molecule (Enneking et al., 2013) unaffected by Myo15 knock-down (Fig. S2, G and H).

Presynaptic mitochondria are essential for synaptic function and, like AZ and SV proteins, require active transport to presynaptic boutons. To assess mitochondrial function upon Myo15 depletion, we analyzed endogenous ATP synthase (ATP5) levels. Since mitochondria also localize to muscles beneath the NMJ (Fig. S2 I), we excluded muscle contributions by masking neuronal membranes with HRP and analyzed only HRP-positive NMJ sections (Fig. S2 J). We detected no changes in mitochondrial area (Fig. S2 K), density (Fig. S2 L), mean pixel intensity of single mitochondria (Fig. S2 M), or total ATP5 signal across the NMJ (Fig. S2 N). This suggests that mitochondrial transport and synaptic anchorage remain unaffected by Myo15 depletion.

Finally, we analyzed early endosomes (EEs) at the synaptic terminal, implicated in SV recycling and presynaptic material distribution (Sudhof, 2004; Jahne et al., 2015; Ivanova and Cousin, 2022). Analogous to mitochondria, EE detection was complicated by their presence in muscles beneath the NMJ, requiring an extended analysis workflow (Fig. 2 L versus Fig. S2 O). At Myo15-depleted NMJs, staining with the EE marker rabenosyn-5 (Rbsn-5, a Rab5 effector (Tanaka and Nakamura, 2008), Fig. 2 L) revealed a ∼15% reduction in single EE area (Fig. 2 M) and a 40% increase in EE density (Fig. 2 N), indicating structural changes of the EE. However, single EE Rbsn-5 mean pixel intensity (Fig. 2 O) remained unchanged compared with controls. Total EE intensity across the NMJ (Fig. 2 P) was not altered but showed a trend toward an increase.

In summary, Myo15 specifically regulates the abundance, availability, and distribution of a subset of organelles and proteins at the synaptic terminal, including mature core AZ scaffold proteins (BRP and RIM-BP), SV components (VGlut and Rab3), and EEs. In contrast, early presynaptic AZ proteins (liprin-α), SV endocytosis regulators (Dap160), cell adhesion proteins (fasciclin 2 and neuroglian), and mitochondria appear unaffected by Myo15 function.

### Elevated Myo15 levels increase AZ size and terminal length

**Figure 3.**
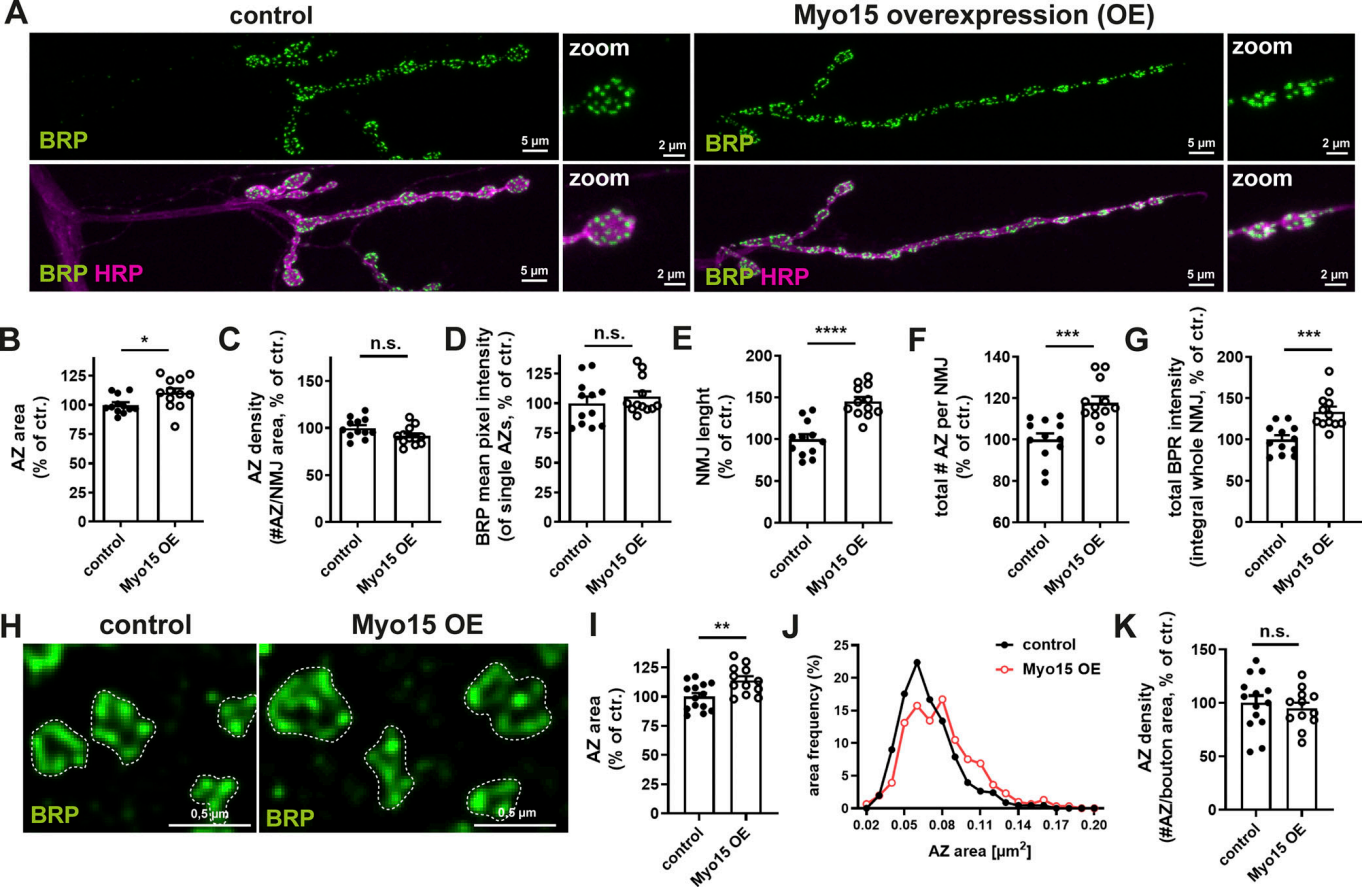
**Elevated Myo15 level increases AZ size and terminal length. (A)** Confocal images of NMJs upon Myo15 overexpression and control (UAS–Gal4 system), stained for BRP (green) and HRP (magenta), scale bar overview 5 µm, zoom 2 µm. **(B–G)** Quantifications of A. **(B)** AZ area (control 100.0 ± 2.24% *n* = 12; Myo15 OE 110.3 ± 3.81%, *n* = 12), (C) density of AZs, as number of AZ per NMJ area (control 100.0 ± 3.04%, *n* = 12; Myo15 OE 91.59 ± 2.82%, *n* = 12), (D) BRP mean pixel intensity of single AZs (control 100.0 ± 5.52%, *n* = 12; Myo15 OE 105.5 ± 4.49%, *n* = 12), (E) NMJ length (control 100.0 ± 6.02%, *n* = 12; Myo15 OE 145.3 ± 5.38%, *n* = 12). **(F)** Total number of all AZs per NMJ (control 100.0 ± 3.00%, *n* = 12; Myo15 OE 117.7 ± 3.14%, *n* = 12). **(G)** Total BRP intensity as integral of the BRP mean pixel intensity over the entire NMJ (control 100.0 ± 4.00%, *n* = 12; Myo15 OE 133.6 ± 6.13%, *n* = 12). **(H)** STED imaging of AZs labeled with BRP (green) upon Myo15 overexpression (UAS–Gal4 system), (scale bar 0.5 µm). **(I–K)** Quantification of H. **(I)** AZ area (control 100.0 ± 3.11%, *n* = 14; Myo15 OE 113.9 ± 3.51%, *n* = 12), (J) relative frequency histogram of AZ areas (total number AZs: control 456 and Myo15 OE 306), bin width 0.01, (K) density of AZs, as number of AZ per NMJ area (control 100.0 ± 6.84%, *n* = 14; Myo15 OE 95.03 ± 5.21%, *n* = 12). All data are provided as mean ± SEM. For B–G: n represents NMJs with 1–2 NMJs/animal from five to six animals, for I–K: n represents boutons, three to four animals with 1–2 NMJs/animal and 1–4 boutons/NMJ, *P < 0.05; **P < 0.01; ***P < 0.001; ****P < 0.0001. OE, overexpression.

Reduced presynaptic Myo15 level causes AZ size reduction and an increase in AZ density, possibly due to a limited availability or distribution of presynaptic material resources. We therefore speculated that increasing Myo15 levels could cause an opposite effect. Using the UAS–Gal4 system, we overexpressed Myo15 and performed confocal BRP imaging (Fig. 3 A). This revealed that AZ size was increased by ∼10% (Fig. 3 B) with unchanged AZ density (Fig. 3 C) and BRP mean intensity per AZ (Fig. 3 D). Notably, NMJ length was increased by nearly 40% (Fig. 3 E), while NMJ shape remained regular without satellite bouton formation. As a result, the total number of AZs and BRP signal intensity summed up over the NMJ increased (Fig. 3, F and G). Next, we examined the AZ scaffold architecture and performed STED microscopy of NMJs overexpressing Myo15 (Fig. 3 H), confirming a 15% AZ area enlargement (Fig. 3, I and J) and an unaltered AZ density (Fig. 3 K).

### Myo15 is present at presynaptic boutons of neuromuscular terminals

**Figure S3.**
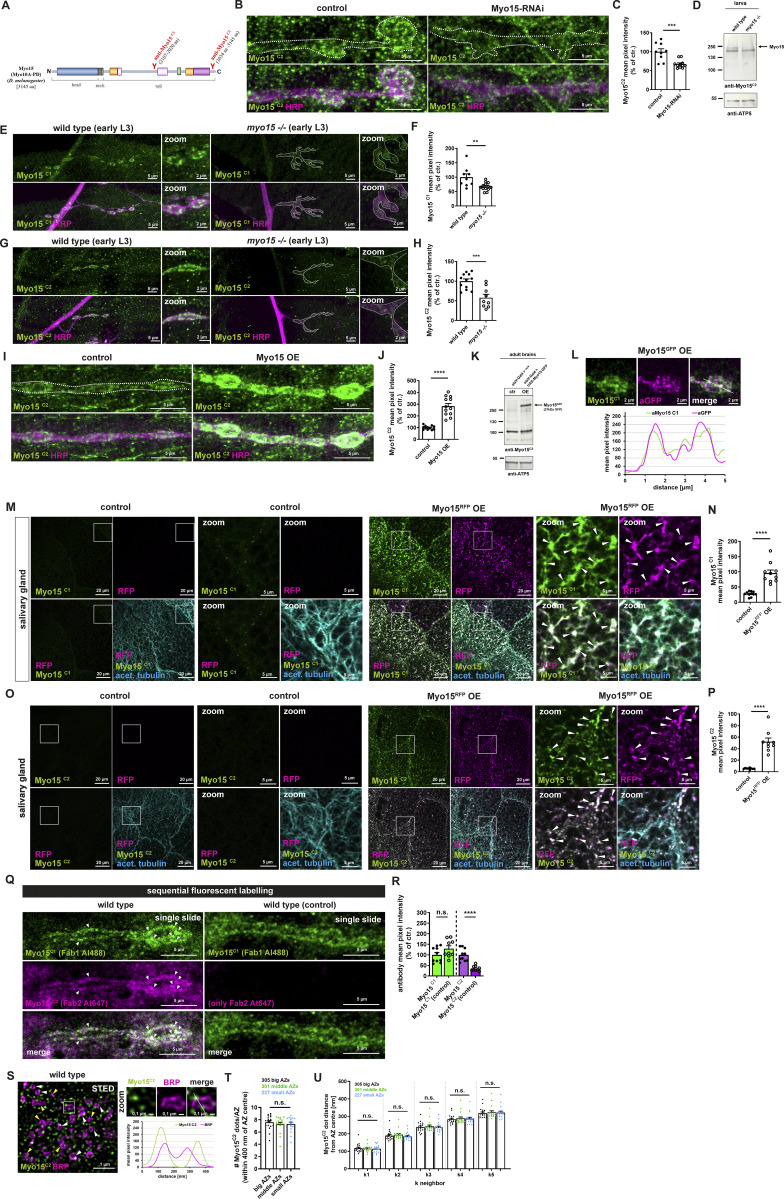
**Myo15 localization at the NMJ and salivary gland and antibody verification. (A)** Schematic representation of *Drosophila* class XV Myo15 indicating antibody position (Myo15^C1^ and Myo15^C2^). **(B)** Confocal images of control and Myo15-RNAi knock-down NMJs stained for Myo15 (anti-Myo15^C2^, green) and HRP (magenta), scale bar overview 5 µm. **(C)** Quantification of B: Myo15^C2^ mean pixel intensity (control 100.0 ± 7.82%, *n* = 9; Myo15-RNAi 67.4 ± 3.24%, *n* = 11). **(D)** Western blot of wild-type and *myo15*^*−/−*^ mutant larvae for Myo15^C2^. **(E)** Confocal images of wild-type and *myo15*^*−/−*^ mutant NMJs stained for Myo15 (anti-Myo15^C1^, green) and HRP (magenta), scale bar overview 5 µm, zoom 2 µm. **(F)** Quantification of E. Myo15^C1^ mean pixel intensity (wild type 100.0 ± 11.52%, *n* = 9; *myo15*^*−/−*^ mutant 67.38 ± 3.79%, *n* = 13). **(G)** Confocal images of wild-type and *myo15*^*−/−*^ mutant NMJs stained for Myo15 (anti-Myo15^C2^, green) and HRP (magenta), scale bar overview 5 µm, zoom 2 µm. **(H)** Quantification of G. Myo15^C2^ mean pixel intensity (wild type 100.0 ± 5.71%, *n* = 12; *myo15*^*−/−*^ mutant 58.05 ± 8.54%, *n* = 9). **(I)** Confocal images of NMJs upon motoneuronal Myo15 overexpression and control, stained for Myo15^C2^ (green) and HRP (magenta), scale bar overview 5 µm. **(J)** Quantification of I: Myo15^C2^ mean pixel intensity (control 100.0 ± 4.89%, *n* = 12; Myo15 OE 282.4 ± 23.21%, *n* = 12). **(K)** Western blot of adult brains neuronally overexpressing Myo15 and control brains for Myo15^C2^. **(L)** Confocal images of control and Myo15^GFP^-OE NMJs co-labeled Myo15 (anti-Myo15^C1^, green) and GFP-tagged Myo15 (magenta), scale bar 2 µm. White line marks the plane of line profile. Lower panel shows the line profile. **(M)** Confocal images of salivary glands overexpressing Myo15 and control, stained for Myo15^C1^ (green), acetylated tubulin (blue); RFP (no antibody staining, magenta), scale bar overview 20 µm, zoom 5 µm. **(N)** Quantification of M. Myo15^C1^ mean pixel intensity (control 27.59 ± 2.36, *n* = 11; Myo15 OE 95.61 ± 10.44, *n* = 10). **(O)** Confocal images of salivary glands overexpressing Myo15 and control, stained for Myo15^C2^ (green), acetylated tubulin (blue); RFP (no antibody staining, magenta), scale bar overview 20 µm, zoom 5 µm. **(P)** Quantification of O: Myo15^C2^ mean pixel intensity (control 5.49 ± 0.43%, *n* = 9; Myo15 OE 52.07 ± 6.36%, *n* = 9). **(Q)** Confocal images (single optical slides) of wild-type NMJs sequentially stained for Myo15^C1^ with Fab1 goat–anti-rabbit Al488 (green) and Myo15^C2^ with Fab1 goat–anti-rabbit Al647 (magenta), for control (right panel) no primary Myo15^C2^ antibody was applied, scale bar 5 µm, white arrowheads mark overlapping signal of both antibodies. **(R)** Quantification of Q: Myo15^C1^/Myo15^C2^ mean pixel intensity (Myo15^C1^/Fab1Al488 100 ± 11.3%, *n* = 10; control 129 ± 13.82%, *n* = 9; Myo15^C2^/Fab1Al647 100 ± 9.47%, *n* = 10; control 35.63 ± 4.05%, *n* = 9). **(S)** STED imaging of wild-type NMJs stained for Myo15 (anti-Myo15^C2^, green) BRP (magenta), scale bar 1 µm, zoom 0.1 µm. White square marks zoom area, white dashed line in zoom marks the plane of line profile. Lower panel shows line profile, green anti-Myo15^C2^, magenta BRP. **(T)** Quantification of S. Number of Myo15 spots within 400 nm of AZ center, binned for small (area = 1–159 pixel^2^, total 227 AZs) with 7.58 ± 0.27 Myo15 dots, middle (160–215 pixel^2^, total 301 AZs) with 7.29 ± 0.29 Myo15 dots, big (<215 pixel^2^, total 305 AZs) with 7.32 ± 0.28 Myo15 dots (1 pixel = 18,9 nm^2^). **(U)** Quantification of S. K-neighbor analysis (k 1–5) determining Myo15 (aMyo15^C2^) dot distance from the AZ center (BRP ring center) comparing small (area = 1–159 pixel^2^, total 227 AZs), middle (160–215 pixel^2^, total 301 AZs), and big (<215 pixel^2^, total 305 AZs) AZs (1 pixel = 18,9 nm^2^). All data are provided as mean ± SEM. **(C, F, H, J, and R)***n* represents NMJs with 1–2 NMJs/animal from five to six animals; N, P: n represents one salivary gland with 1/2 salivary glands per animal from five to six animals, T: n = image, with 1–2 boutons/image and 3 images/NMJ, 2 NMJs/animal, three to four animals in total. ****P < 0.0001; ***P < 0.001; **P < 0.01; *P < 0.05; n.s., not significant. OE, overexpression. Source data are available for this figure: SourceData FS3.

**Figure 4.**
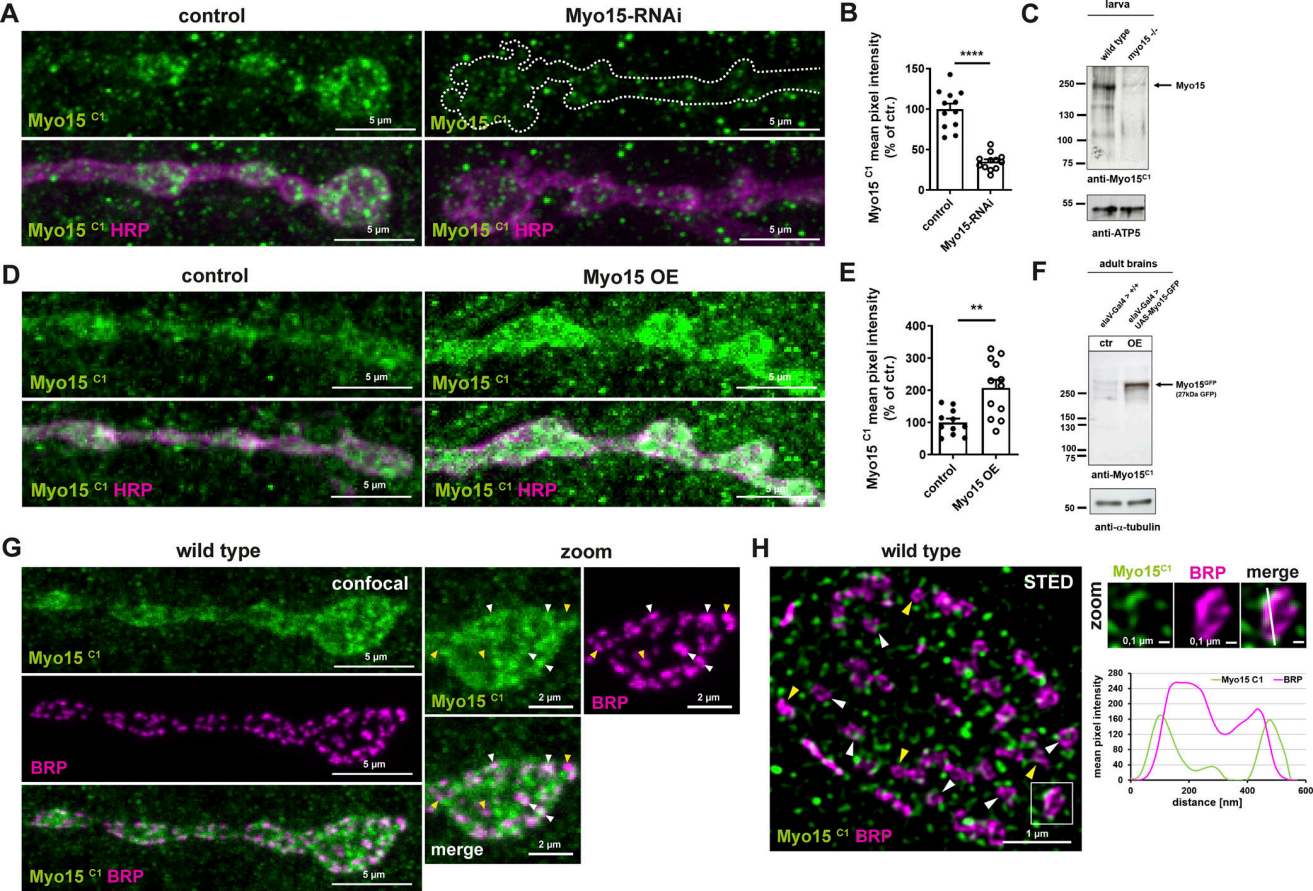
**Myo15 localizes to the presynaptic bouton. (A)** Confocal images of control and Myo15-RNAi knock-down NMJs stained for Myo15 (anti-Myo15^C1^, green) and HRP (magenta), scale bar 5 µm. **(B)** Quantification of A. Myo15^C1^ mean pixel intensity (control 100.0 ± 6.89%, *n* = 12; Myo15-RNAi 35.08 ± 3.0%, *n* = 12). **(C)** Western blot of wild-type and *myo15*^*−/−*^ mutant larva for Myo15^C1^. **(D)** Confocal images of NMJs upon motoneuronal Myo15 overexpression and control, stained for Myo15^C1^ (green) and HRP (magenta), scale bar 5 µm. **(E)** Quantification of D. Myo15^C1^ mean pixel intensity (control 100.0 ± 11.99%, *n* = 11; Myo15 OE 207.6 ± 25.55%, *n* = 12). **(F)** Western blot of adult brains neuronally overexpressing Myo15 and control brains for Myo15^C1^. **(G)** Confocal image of wild-type NMJ stained for Myo15 (anti-Myo15^C1^, green) and BRP (magenta), scale bar overview 5 µm, zoom 2 µm. White arrowheads mark Myo15 accumulations near or at an AZ, yellow arrowheads mark AZ without Myo15 accumulations. **(H)** STED imaging of wild-type NMJs stained for Myo15 (anti-Myo15^C1^, green) and BRP (magenta), scale bar overview 1 µm, zoom 0.1 µm. White square marks the zoom area, the white dashed line in the zoom marks the plane of line profile. Lower panel shows line profile, arrowheads as in G. All data are provided as mean ± SEM. **(B and E)***n* represents NMJs with 1–2 NMJs/animal from five to six animals, ****P < 0.0001; ***P < 0.001; **P < 0.01; *P < 0.05; n.s., not significant. OE, overexpression. Source data are available for this figure: SourceData S4.

Next, we wanted to assess the distribution of endogenous Myo15 protein and generated two Myo15 antibodies targeting the C-terminal tail (Fig. S3 A). Myo15^C1^ recognizes a sequence C-terminal to the split FERM-like domain (aa 2107–2020) and detects the three large Myo15 isoforms but not the short PE isoform, while Myo15^C2^ targets a region N-terminal of the MyTH4-FERM tandem (aa 3034–3145) and recognizes all four Myo15 isoforms. Both antibodies labeled NMJ boutons in a cloudy, partially dot-like pattern, and we also observed a dotted muscle labeling, stronger with the Myo15^C2^ antibody, possibly due to detection of the short PE isoform, which Myo15^C1^ does not recognize (Fig. 4 A and Fig. S3 B).

Antibody validation was performed using multiple approaches. First, immunofluorescence staining of NMJs of Myo15-RNAi–mediated knock-down animals showed a ∼60% reduction of Myo15^C1^ (Fig. 4, A and B) and a ∼40% reduction of Myo15^C2^ signal (Fig. S3, B and C), consistent with the RNAi efficacy from our Myo15-RFP depletion assay (Fig. S1, B and C). Due to the observed muscle contribution, we applied the extended imaging analysis workflow similar to the mitochondria and EE analysis (Fig. S2, H, I, and N). The residual Myo15 signal reflects the hypomorphic RNAi knock-down of the zygotic and persisting maternal myosin contribution.

Second, we performed western blot analysis of wild-type and *myo15*^*−/−*^ mutant larvae. In the wild type, both antibodies detected a ∼250-kDa band, consistent with previous reports (Liu et al., 2008), which was strongly reduced in *myo15*^*−/−*^ mutants but still present at low levels, possibly due to maternal contribution (Fig. 4 C and Fig. S3 D). Myo15^C2^ also detected an additional ∼220-kDa band, unaffected in mutant animals, indicating possible cross-reactivity with another protein (Fig. S3 D).

Third, we validated the antibodies via immunofluorescence analysis in wild-type and *myo15*^*−/−*^ NMJs, where signal intensity was reduced by ∼30% for Myo15^C1^ and ∼40% for Myo15^C2^ (Fig. S3, E–H). Given the stronger RNAi-mediated reduction, this further supports the idea that maternal contribution possibly accounts for the residual Myo15 signal in mutants.

Additionally, we tested both antibodies in an overexpression approach using the UAS–Gal4 system. This resulted in a two- to threefold increase in Myo15 signal at NMJs (Fig. 4, D and E; and Fig. S3, I and J). Western blot analysis of adult fly brains neuronally overexpressing Myo15-GFP revealed an increased ∼280-kDa band compared with control, matching the predicted Myo15-GFP fusion protein (Fig. 4 F and Fig. S3 K), confirming antibody specificity. Myo15^C2^ detected an unchanged additional ∼100-kDa band, likely an unspecific interaction. Further co-labeling of Myo15 antibodies with GFP-tagged Myo15 in NMJs showed co-localization of both signals, supporting antibody specificity (Fig. S3 L). Additionally, overexpression of Myo15-RFP in salivary glands, where muscle staining is absent, resulted in a four- to ninefold signal increase for both antibodies and Myo15-RFP, and the antibody signal overlapped in the zoom images, further confirming specificity (Fig. S3, M–P).

To directly compare Myo15^C1^ and Myo15^C2^ labeling, we performed sequential labeling with differentially tagged Fab fragments using Fab fragment blocking (see Materials and methods, [Gimber et al., 2022]). Both antibodies co-localized at NMJs and partially in muscles, while controls lacking Myo15^C2^ showed no specific signal (Fig. S3, Q and R).

After antibody validation, we performed Myo15 co-stainings with BRP at the NMJ and observed that Myo15 is enriched in presynaptic boutons, with a cloudy, partially dot-like pattern and less signal in inter-bouton regions (Fig. 4 G). In confocal and STED imaging, we observed that Myo15 localizes adjacent to and directly at AZs (Fig. 4, G and H; and Fig. S3 S, white arrowheads), partially near the planar BRP ring (Fig. 4 H and Fig. S3 S zooms). Not all AZs were decorated with Myo15 puncta (yellow arrowheads), and some Myo15 puncta appeared in the bouton cytoplasm between AZs.

K-neighbor analysis revealed that the amount of Myo15 dots at an AZ was independent of the AZ size (Fig. S3 T) and equally that the distance between a Myo15 dot (k1–k5) and the AZ center was not correlated with AZ size (binned in small, middle, and large AZs, Fig. S3 U).

### Myo15 partially co-localizes with and regulates actin at the synaptic terminal

**Figure 5.**
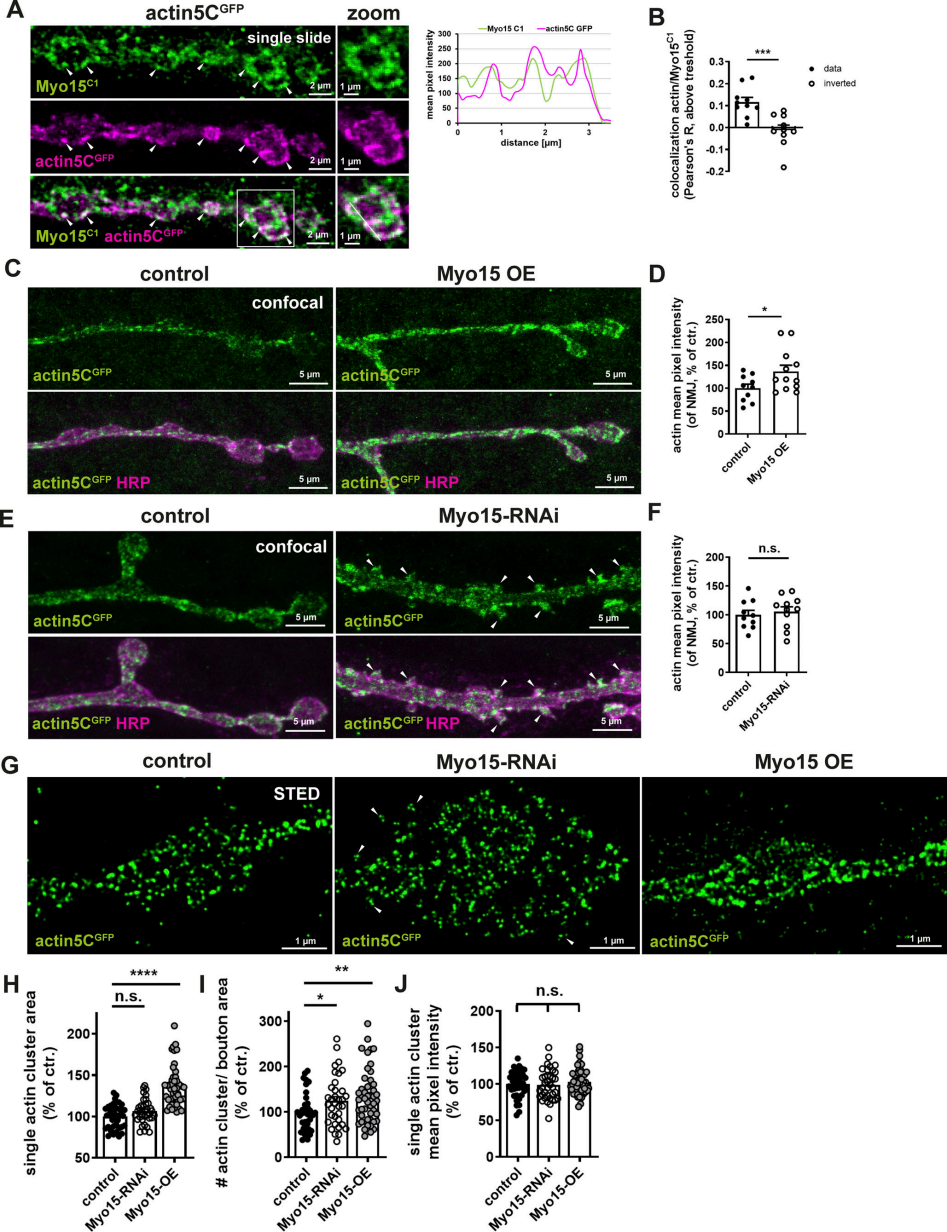
**Myo15 partially co-localizes with and regulates actin at the synaptic terminal. (A)** Single optical slide of confocal images of NMJs expressing actin5C^GFP^ using the UAS–Gal4 expression system, stained for Myo15^C1^ (green) and GFP (actin, magenta), scale bar overview 2 µm, zoom 1 µm, white arrowheads mark overlapping Myo15 and actin signals. White square marks the zoom area, white line in zoom marks the plane of line profile. Right panel shows line profile, green Myo15^C1^, magenta actin5C^GFP^. **(B)** Quantification of A. Pearson’s R value of Myo15^C1^ and actin5C^GFP^ (Pearson’s R above threshold 0.1159 ± 0.0211; inverted images −0.0124 ± 0.0236, *n* = 10). **(C)** Confocal images of NMJs expressing actin5C^GFP^, Myo15 (OE), and control stained for GFP (actin, green) and HRP (magenta), scale bar 5 µm. **(D)** Quantification of (C), actin mean pixel intensity at the NMJ (control 100.0 ± 9.06%, *n* = 10; Myo15 OE 136.9 ± 13.24%, *n* = 12). **(E)** Confocal images of NMJs expressing actin5C^GFP^, Myo15-RNAi, and control stained for GFP (actin, green) and HRP (magenta), white arrowheads mark actin accumulations in ectopic satellite boutons, scale bar 5 µm. **(F)** Quantification of E. actin mean pixel intensity at the NMJ (control 100.0 ± 7.88%, *n* = 10; Myo15-RNAi 105.5 ± 8.38%, *n* = 11). **(G)** STED imaging of NMJs expressing actin5C^GFP^ (control) and Myo15-RNAi or Myo15 (OE) labeled for actin (green), white arrowheads mark actin accumulations in ectopic satellite boutons, scale bar 1 µm. **(H–J)** Quantification of G. **(H)** Single actin cluster area (control 100.0 ± 2.21%, *n* = 41; Myo15-RNAi 106.4 ± 2.51%, *n* = 35; Myo15 OE 136.9 ± 3.69%, *n* = 45), (I) actin cluster density as the number of actin clusters per bouton area (control 100.0 ± 6.81%, *n* = 37; Myo15-RNAi 124.5 ± 9.39%, *n* = 35; Myo15 OE 134.0 ± 8.63%, *n* = 47), (J) single actin cluster mean pixel intensity (control 100.0 ± 2.8%, *n* = 41; Myo15-RNAi 99.07 ± 3.41%, *n* = 37; Myo15 OE 103.1 ± 2.60%, *n* = 47). All data are provided as mean ± SEM. **(D and F)***n* represents NMJs with 1–2 NMJs/animal from five to six animals, G: *n* = image, with 1–2 boutons/image and 3 images/NMJ, 2 NMJs/animal, three to four animals in total, ****P < 0.0001; ***P < 0.001; **P < 0.01; *P < 0.05; n.s., not significant. OE, overexpression.

Myo15 contains an actin-binding domain in its head region, enabling its association with the presynaptic actin meshwork (Kneussel and Wagner, 2013). To visualize actin, we overexpressed actin5C^GFP^ and co-labeled for Myo15, revealing a partial overlap of both signals, indicating that a fraction of Myo15 localized to the local actin meshwork at the NMJ (Fig. 5 A, line graph, Fig. 5 B, Pearson’s R value 0,12 was significantly higher compared to the control, using inverted images, Pearson’s R −0.012). Notably, not all actin was co-labeled with Myo15, and not all Myo15 dots localized to actin, as indicated by the comparatively low Pearson’s R value.

Myo15 was previously reported to affect actin stability in *Drosophila* bristle formation (Rich et al., 2021). To test whether Myo15 also regulates actin dynamics at the synaptic terminal, we altered Myo15 levels at the NMJ and monitored actin5C^GFP^ using confocal microscopy. Myo15 overexpression caused a 40% increase in actin signal across the NMJ (Fig. 5, C and D) and Myo15 depletion caused no change in overall actin intensity (Fig. 5, E and F). Instead, it resulted in a strong actin accumulation in additionally forming satellite boutons (Fig. 5 E, arrowheads), a phenotype neither observed in controls nor overexpression animals. Consistently, also STED microscopy revealed a 50% increase in actin cluster size upon Myo15 overexpression and an increase in actin cluster density (number of actin clusters/bouton area, Fig. 5 I), while single actin cluster mean pixel intensity remained unaltered (Fig. 5 J), suggesting the formation of a larger, more compacted actin meshwork. Myo15 depletion did not alter actin cluster size, but actin filament density was increased by ∼25%, possibly indicating actin fragmentation, while actin cluster mean pixel intensity remained unchanged (Fig. 5, H–J). These findings suggest that Myo15 locally regulates actin meshwork architecture and distribution at the synaptic terminal.

### The MT-binding MyTH4 domains are required for Myo15 function

Myosins acquire functional specificity through a unique set of cargo-binding domains in their tail regions (O’Connell et al., 2007; Lu et al., 2014; Li et al., 2016). The *Drosophila* Myo15 contains the conserved tail domains of class XV myosins (Fig. 1 A). To investigate the functional relevance of these domains, we created a series of genomic *myo15* domain deletions using a CRISPR-Cas9 approach, deleting (1) the FERM domain (PD isoform, aa 2814–3033), (2) the first MyTH4(1) domain (aa 1061–117, including the immediately following first part of the F1 lobe of the FERM-like domain [Rich et al., 2021] [for details see Materials and methods]), and (3) both MyTH4 domains (MyTH4(1) and MyTH4(2), aa 2702–2810). We focused on predicted tail domains ([Liang et al., 1999; Lu et al., 2014; Planelles-Herrero et al., 2016; Rich et al., 2021], predicted by a simple modular architecture research tool or conserved domain database [Rich et al., 2021]).

**Figure S4.**
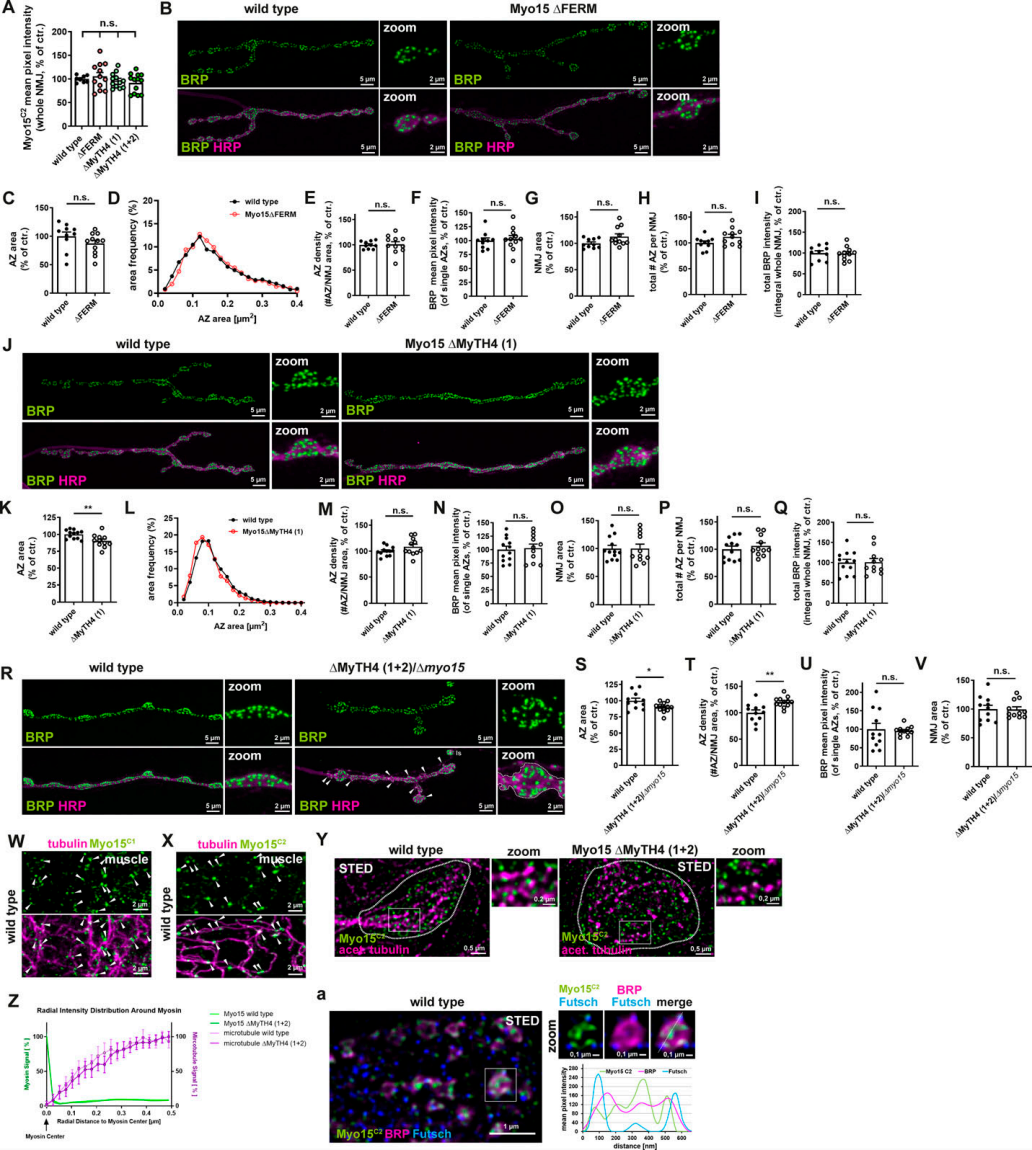
**MyTH4 (1) and FERM domain deletion mutants and Myo15 localization to presynaptic MTs. (A)** Quantification of Myo15^C2^ protein level in deletion mutants: mean pixel intensity wild type 100.0 ± 2.57%, *n* = 8; *Myo15*^ΔFERM^ 105.6 ± 8.06%, *n* = 12, *Myo15*^ΔMyTH4 (1)^ 96.78 ± 4.55%, *n* = 12, *Myo15*^ΔMyTH4 (1+2)^ 91.63 ± 5.58%, *n* = 13). **(B)** Confocal images of *Myo15*^ΔFERM^ deletion mutant and wild-type NMJs stained for BRP (green) and HRP (magenta), scale bar overview 5 µm, zoom 2 µm. **(C–I)** Quantification of B. **(C)** AZ area (control 100.0 ± 7.48% *n* = 10; *Myo15*^ΔFERM^ 87.43 ± 5.61%, *n* = 11). **(D)** Relative frequency histogram of AZ areas (total number of AZs: control 2675 and *Myo15*^ΔFERM^ 3296), bin width 0.02. **(E)** Density of AZs, as number of AZ per NMJ area (control 100.0 ± 2.37% *n* = 10; *Myo15*^ΔFERM^ 101.1 ± 5.35%, *n* = 11). **(F)** BRP mean pixel intensity of single AZs (control 100.0 ± 4.83% *n* = 10; *Myo15*^ΔFERM^ 103.1 ± 6.15%, *n* = 11). **(G)** NMJ area (control 100.0 ± 2.84% *n* = 10; *Myo15*^ΔFERM^ 112.8 ± 5.31%, *n* = 11). **(H)** Total number of all AZs per NMJ (control 100.0 ± 3.86% *n* = 10; *Myo15*^ΔFERM^ 112.0 ± 4.90%, *n* = 11). **(I)** Total BRP intensity as integral of the BRP mean pixel intensity over the entire NMJ (control 100.0 ± 5.56% *n* = 10; *Myo15*^ΔFERM^ 98.93 ± 4.94%, *n* = 11). **(J)** Confocal images of *Myo15*^ΔMyTH4 (1)^ deletion mutant and wild-type NMJs stained for BRP (green) and HRP (magenta), scale bar overview 5 µm, zoom 2 µm. **(K–Q)** Quantification of J. **(K)** AZ area (control 100.0 ± 1.86% *n* = 12; *Myo15*^ΔMyTH4 (1)^ 90.21 ± 2.86%, *n* = 11). **(L)** Relative frequency histogram of AZ areas (total number of AZs control 4038 and *Myo15*^DMyTH4 (1)^ 3928), bin width 0.02. **(M)** Density of AZs, as number of AZ per NMJ area (control 100.0 ± 2.55% *n* = 12; *Myo15*^ΔMyTH4 (1)^ 108.5 ± 5.02%, *n* = 11). **(N)** BRP mean pixel intensity of single AZs (control 100.0 ± 6.49% *n* = 12; *Myo15*^ΔMyTH4 (1)^ 102.9 ± 7.54%, *n* = 11). **(O)** NMJ area (control 100.0 ± 5.95% *n* = 12; *Myo15*^ΔMyTH4 (1)^ 100.2 ± 7.90%, *n* = 11). **(P)** Total number of all AZs per NMJ (control 100.0 ± 5.79% *n* = 12; *Myo15*^ΔMyTH4 (1)^ 106.1 ± 5.70%, *n* = 11). **(Q)** Total BRP intensity as integral of the BRP mean pixel intensity over the entire NMJ (control 100.0 ± 8.20% *n* = 12; *Myo15*^ΔMyTH4 (1)^ 100.8 ± 9.17%, *n* = 11). **(R)** Confocal images of *Myo15*^*ΔMyTH4* (*1+2*)^*/Δmyo15* trans-heterozygous mutant and wild-type NMJs stained for BRP (green) and HRP (magenta), scale bar overview 5 µm, zoom 2 µm. **(S–V)** Quantification of R. **(S)** AZ area (control 100.0 ± 3.90% *n* = 11; *Myo15*^*ΔMyTH4* (*1+2*)^*/Δmyo15* 89.71 ± 2.17%, *n* = 12). **(T)** Density of AZs, as number of AZ per NMJ area (control 100.0 ± 5.45% *n* = 11; *Myo15*^*ΔMyTH4* (*1+2*)^*/Δmyo15* 120.4 ± 2.82%, *n* = 12). **(U)** BRP mean pixel intensity of single AZs (control 100.0 ± 15.98% *n* = 11; *Myo15*^*ΔMyTH4* (*1+2*)^*/Δmyo15* 95.75 ± 3.85%, *n* = 11). **(V)** NMJ area (control 100.0 ± 6.61% *n* = 11; *Myo15*^*ΔMyTH4* (*1+2*)^*/Δmyo15* 99.32 ± 4.6%, *n* = 11). **(W and X)** Confocal images of wild-type muscles stained for (W) Myo15^C1^ (green) or (X) Myo15^C2^ (green) and acetylated tubulin (magenta), white arrowheads mark Myo15 spots localizing to MTs, scale bar 2 µm. **(Y)** STED images of wild-type and *Myo15*^ΔMyTH4 (1+2)^ deletion mutant stained for Myo15^C2^ (green), MTs (acetylated tubulin, magenta), scale bar overview 0.5 µm, zoom 0.2 µm. White square mark zoom area. **(Z)** Quantification of Y. Normalized radial intensity distribution of MT and myosin signal around the centers of myosin clusters for *Myo15*^ΔMyTH4 (1+2)^ deletion mutant and wild type. Green as control compares Myo15 to Myo15 distances in both phenotypes, magenta compares Myo15 to MT distance. Means ± 95% confidence intervals (dashed lines) from 25/28 (wild type/mutant) images are shown. **(a)** STED imaging of wild-type NMJs stained for Myo15^C2^ (green), MTs (futsch, blue), BRP (magenta), bouton scale bar 1 µm, zoom 0.1 µm. White square indicates zoom region, white dashed line in zoom marks the plane of line profile. Lower panel shows line profile, green Myo15^C2^, blue futsch, magenta BRP. All data are provided as mean ± SEM. **(A, C–I, K–Q, and S–V)***n* = NMJs with 1–2 NMJs/animal, four to six animals, Z: *n* = image, with 1–2 boutons/image and 2–3 images/NMJ, 2 NMJs/animal, three to four animals in total****P < 0.0001; ***P < 0.001; **P < 0.01; *P < 0.05; n.s., not significant.

Confocal imaging of Myo15 antibody staining confirmed that all deletion mutants expressed Myo15 at about wild-type levels (Fig. S4 A), indicating stability of the domain-deleted proteins. Deletion of the C-terminal FERM domain produced no detectable synaptic phenotype (Fig. S4, B–I), while deletion of MyTH4(1) caused mild defects, including a slight AZ size reduction (Fig. S4, K and L) but no changes in AZ density, BRP intensity, NMJ length, or total AZ number (Fig. S4, M–Q). Also NMJ morphology remained largely unaffected (Fig. S4 J).

**Figure 6.**
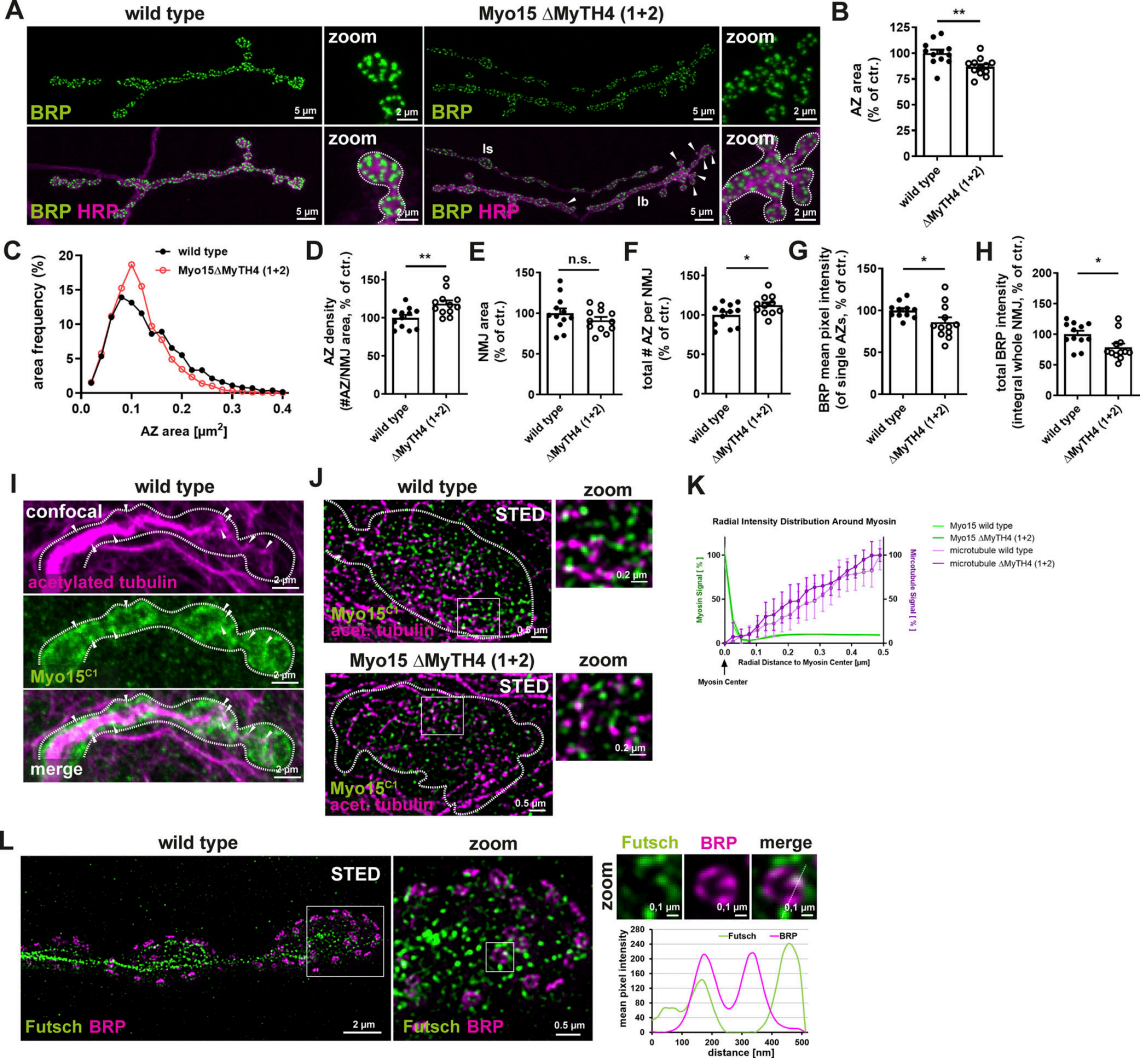
**The MyTH4 domain is required for Myo15 function, and Myo15 partially localizes to presynaptic MTs. (A)** Confocal images of *Myo15*^ΔMyTH4 (1+2)^ deletion mutant and wild-type NMJs stained for BRP (green) and HRP (magenta), scale bar overview 5 µm, zoom 2 µm, white arrowheads mark satellite boutons, Is and Ib NMJs indicated. **(B–H)** Quantification of A. **(B)** AZ area (control 100.0 ± 3.32% *n* = 12; *Myo15*^ΔMyTH4 (1+2)^ 86.71 ± 2.47%, *n* = 12). **(C)** Relative frequency histogram of AZ areas (total number of AZs: control 3864 and *Myo15*^ΔMyTH4 (1+2)^ 4220), bin width 0.02. **(D)** Density of AZs, as number of AZ per NMJ area (control 100.0 ± 3.83%, *n* = 12; *Myo15*^ΔMyTH4 (1+2)^ 118.3 ± 4.52%, *n* = 12). **(E)** NMJ area (control 100.0 ± 5.92%, *n* = 12; *Myo15*^ΔMyTH4 (1+2)^ 91.46 ± 4.07%, *n* = 12). **(F)** Total number of all AZs per NMJ (control 100.0 ± 4.05%, *n* = 12; *Myo15*^ΔMyTH4 (1+2)^ 112.2 ± 3.76%, *n* = 11). **(G)** BRP mean pixel intensity of single AZs (control 100.0 ± 2.84%, *n* = 12; *Myo15*^ΔMyTH4 (1+2)^ 86.09 ± 5.80%, *n* = 12). **(H)** Total BRP intensity as integral of the BRP mean pixel intensity over the entire NMJ (control 100.0 ± 5.61% *n* = 12; *Myo15*^ΔMyTH4 (1+2)^ 78.61 ± 6.19%, *n* = 12). **(I)** Confocal images of wild-type NMJs stained for MTs (acetylated tubulin, magenta) and Myo15^C1^ (green), scale bar overview 2 µm, white arrowheads mark overlapping Myo15 and MT signal. **(J)** STED images of wild-type and *Myo15*^ΔMyTH4 (1+2)^ deletion mutant stained for Myo15^C1^ (green), MTs (acetylated tubulin, magenta), scale bar overview 0.5 µm, zoom 0.2 µm. White square mark zoom area. **(K)** Quantification of J. Normalized radial intensity distribution of MT and myosin signal around the centers of myosin clusters for *Myo15*^ΔMyTH4 (1+2)^ deletion mutant and wild type. Green as control compares Myo15 to Myo15 distances in both phenotypes, magenta compares Myo15 to MT distance. Means ± 95% confidence intervals (dashed lines) from 23/18 (wild type/mutant) images are shown. **(L)** STED imaging of wild-type NMJs stained for the MAP1 homolog futsch, localizing to MTs (green) and BRP (magenta), scale bar overview 2 µm, zoom 0.5 and 0.1 µm. White square mark zoom area, white dashed line in zoom marks the plane of line profile. Lower panel shows line profile, green futsch, magenta BRP. All data are provided as mean ± SEM. **(B–H)**: *n* represents NMJs with 1–2 NMJs/animal from five to six animals, K: *n* = image, with 1–2 boutons/image and 2–3 images/NMJ, 2 NMJs/animal, three to four animals in total. ****P < 0.0001, ***P < 0.001, **P < 0.01, *P < 0.05, n.s. not significant.

In contrast, simultaneous deletion of both MyTH4(1+2) domains caused a phenotype similar to Myo15 knock-down, including irregular NMJ morphology, unevenly shaped boutons, and satellite bouton formation (Fig. 6 A, arrowheads). This phenotype was accompanied by a decrease in AZ size (Fig. 6, B and C) and an increase in AZ density (Fig. 6 D). As seen in knock-down animals, the NMJ area remained unchanged (Fig. 6 E) and the total AZ number increased (Fig. 6 F). Additionally, BRP intensity per AZ slightly decreased, leading to a modest reduction in total BRP protein levels at the NMJ (Fig. 6, G and H).

To confirm the critical role of MyTH4 domains for Myo15 function, we analyzed the MyTH4(1+2) deletion allele in trans to the *myo15*^*−/−*^ mutant allele. Trans-heterozygous animals exhibited similar NMJ defects as homozygous mutants, including irregular NMJs, satellite boutons, and thickened inter-bouton regions (Fig. S4 R, arrowheads). Similarly, the AZ area was reduced (Fig. S4 S), AZ density was increased (Fig. S4 T), and BRP intensity per AZ as well as NMJ area remained unchanged (Fig. S4, U and V). Since the trans-heterozygous phenotype was not more severe than the *myo15*^*−/−*^ homozygous mutant, we conclude that the MyTH4(1+2) deletion functions similarly to a null allele, confirming that these domains are essential for Myo15 function at the presynaptic terminal.

### Myo15 localizes to presynaptic MTs

The MyTH4 domain was shown to directly bind MTs (Weber et al., 2004; Hirano et al., 2011), suggesting that Myo15 function may depend on MT interactions. To test this hypothesis, we performed tubulin co-staining at the synaptic terminal and found Myo15 localizing to a subset of acetylated tubulin clusters in confocal images (Fig. 6 I, arrowheads). More distinctly than at the NMJ, where the underlying muscle also contained MTs, Myo15 localized to MTs in muscle tissue (Fig. S4, W and X, arrowheads) and decorated MTs in salivary glands when Myo15-RFP was ectopically expressed (Fig. S3, M and O, zooms).

At STED resolution, Myo15 frequently accumulated in patches near MTs traversing the bouton (Fig. 6 J, upper panel; Fig. S4 Y, left panel). To assess whether Myo15 localization to MT depends on the MyTH4 domains, we compared the distance distribution of Myo15 to MTs in wild-type and *Myo15*^ΔMyTH4 (1+2)^ deletion mutant. Radial profile analysis (Schmerl et al., 2022) (https://github.com/ngimber/ClusterProfile) of Myo15^C1^ and Myo15^C2^ relative to acetylated tubulin (Fig. 6, J and K; and Fig. S4, Y and Z) showed no significant change in Myo15-MT distance. However, given that only a fraction of Myo15 molecules are localized to MTs, this analysis might be hampered. At STED resolution, thick MT bundles marked by futsch (MAP1 homolog) (Hummel et al., 2000) spanned the entire bouton, while thinner bundles bypassed individual AZs (Fig. 6 L). Triple labeling of AZ scaffolds, MTs, and Myo15 revealed a subset of AZs where Myo15 overlapped with or was adjacent to the futsch/MT signal (Fig. S4 a).

### Myo15 is required for structural and functional PHP

**Figure S5.**
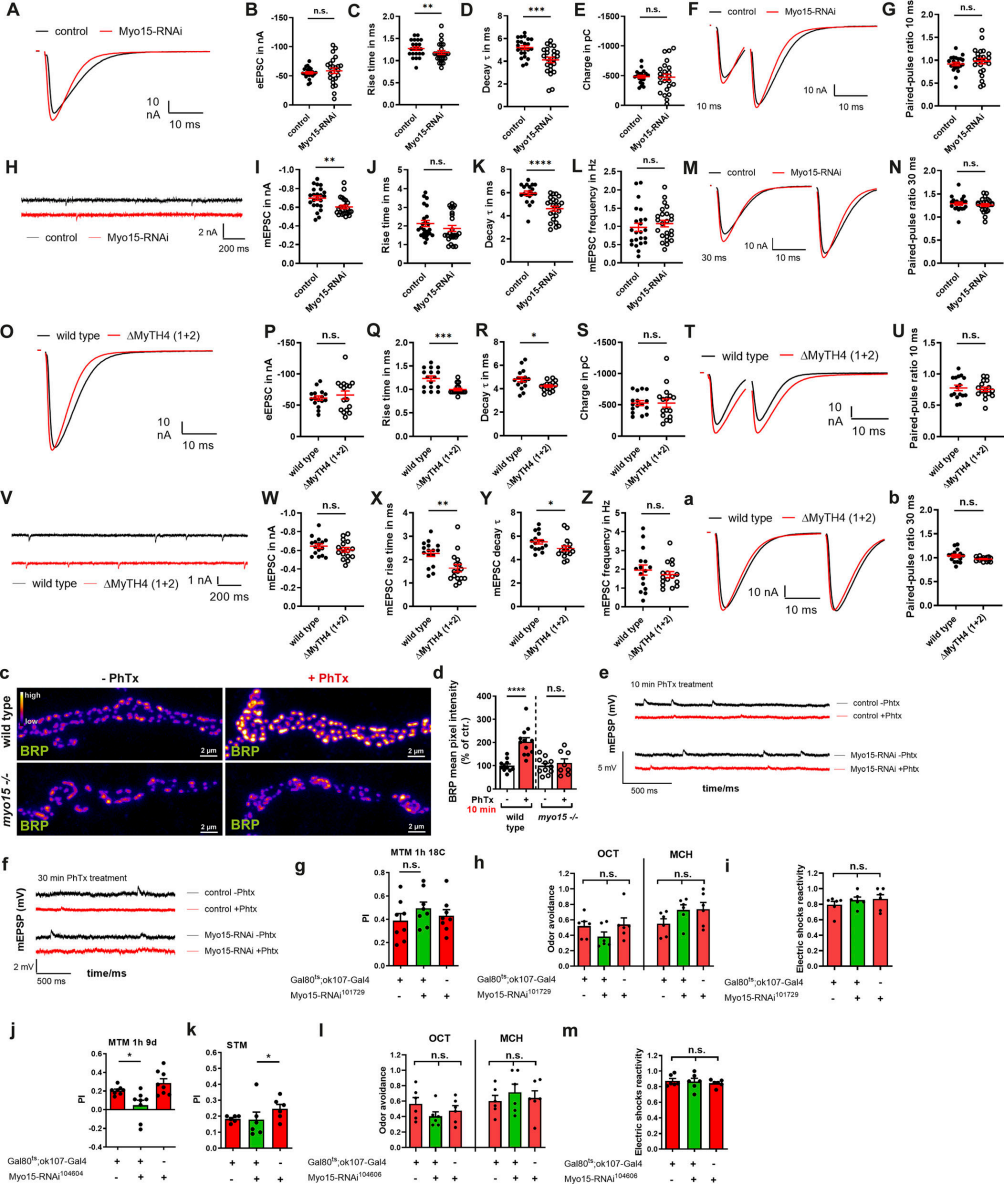
**Electrophysiological TEVC analysis of Myo15 loss-of-function, PhTx treatment of *Myo15*** ^***−/−***^ **mutant, and sustained memory consolidation for Myo15 knock-down.** **(A–N)** TEVC electrophysiological recordings of Myo15 RNAi–mediated knock-down, (A–G, M, and N) evoked, (H–L) spontaneous release. **(A)** eEPSC traces of Myo15 knock-down (red) and control (black) NMJs, scale bar 10 nA, 10 ms. **(B)** eEPSC amplitude (control −55.43 ± 2.00 nA, *n* = 22; Myo15-RNAi −58.92 ± 4.71 nA, *n* = 24). **(C)** Rise time (control 1.27 ± 0.04 ms, *n* = 22; Myo15-RNAi 1.18 ± 0.04 ms, *n* = 24). **(D)** Decay time τ (control 5.2 ± 0.17 ms, *n* = 23; Myo15-RNAi 4.11 ± 0.24 ms, *n* = 24). **(E)** Charge (control −484.5 ± 23.03 pC, *n* = 22; Myo15-RNAi −476.4 ± 52.17 pC, *n* = 24). **(F)** 10-ms paired-pulse traces of Myo15 knock-down (red) and control (black) NMJs, scale bar 10 nA, 10 ms. **(G)** Paired-pulse ratio 10 ms (control 0.92 ± 0.04 ms, *n* = 22; Myo15-RNAi 0.98 ± 0.06, *n* = 24). **(H)** mEPSC traces of Myo15 knock-down (red) and control (black) NMJs, scale bar 2 nA, 200 ms. **(I)** mEPSC amplitude (control −0.70 ± 0.02 nA, *n* = 23; Myo15-RNAi −0.60 ± 0.02 nA, *n* = 24). **(J)** mEPSC rise time (control 2.13 ± 0.16 ms, *n* = 23; Myo15-RNAi 1.87 ± 0.16 ms, *n* = 24). **(K)** mEPSC decay time τ (control 5.95 ± 0.19 ms, *n* = 19; Myo15-RNAi 4.61 ± 0.21 ms, *n* = 23). **(L)** mEPSC frequency (control 0.97 ± 0.12 Hz, *n* = 23; Myo15-RNAi 1.08 ± 0.09 Hz, *n* = 24). **(M)** 30 ms paired-pulse traces of Myo15 knock-down (red) and control (black) NMJs, scale bar 10 nA, 10 ms. **(N)** Paired-pulse ratio 30 ms (control 1.29 ± 0.03 ms, *n* = 22; Myo15-RNAi 1.26 ± 0.03, *n* = 24). **(O–b)** TEVC electrophysiological recordings of *Myo15*^ΔMyTH4 (1+2)^ deletion mutant and wild-type NMJs, (O–U, a, and b) evoked, (V–Z) spontaneous release. **(O)** eEPSC traces of *Myo15*^ΔMyTH4 (1+2)^ deletion mutant (red) and wild-type (black) NMJs (black), scale bar 10 nA, 10 ms. **(P)** eEPSC amplitude (wild type −61.25 ± 3.48 nA, *n* = 16; *Myo15*^ΔMyTH4 (1+2)^ −66.16 ± 6.46 nA, *n* = 16). **(Q)** Rise time (wild type 1.23 ± 0.05 ms, *n* = 16; *Myo15*^ΔMyTH4 (1+2)^ 1.00 ± 0.03 ms, *n* = 16). **(R)** Decay time t (wild type 4.80 ± 0.21 ms, *n* = 16; *Myo15*^ΔMyTH4 (1+2)^ 4.23 ± 0.10 ms, *n* = 16). **(S)** Charge (wild type −526.4 ± 39.29 pC, *n* = 16; *Myo15*^ΔMyTH4 (1+2)^ −527.2 ± 67.47 pC, *n* = 16). **(T)** 10 ms paired-pulse traces of *Myo15*^ΔMyTH4 (1+2)^ deletion mutant (red) and wild-type (black) NMJs (black), scale bar 10 nA, 10 ms. **(U)** Paired-pulse ratio 10 ms (wild type 0.78 ± 0.04 ms, *n* = 16; *Myo15*^ΔMyTH4 (1+2)^ 0.75 ± 0.03, *n* = 16). **(V)** mEPSC traces of *Myo15*^ΔMyTH4 (1+2)^ deletion mutant (red) and wild-type (black) NMJs (black), scale bar 1 nA, 200 ms. **(W)** mEPSC amplitude (wild type −0.65 ± 0.02 nA, *n* = 16; *Myo15*^ΔMyTH4 (1+2)^ −0.61 ± 0.02 nA, *n* = 16). **(X)** mEPSC rise time (wild type 2.27 ± 0.13 ms, *n* = 16; *Myo15*^ΔMyTH4 (1+2)^ 1.64 ± 0.16 ms, *n* = 16). **(Y)** mEPSC decay time t (wild type 5.53 ± 0.19 ms, *n* = 16; *Myo15*^ΔMyTH4 (1+2)^ 4.96 ± 0.21 ms, *n* = 16). **(Z)** mEPSC frequency (wild type 1.96 ± 0.27 Hz, *n* = 16; *Myo15*^ΔMyTH4 (1+2)^ 1.72 ± 0.16 Hz, *n* = 16). **(a)** 30-ms paired-pulse traces of *Myo15*^ΔMyTH4 (1+2)^ deletion mutant (red) and wild-type (black) NMJs (black), scale bar 10 nA, 10 ms. **(b)** Paired-pulse ratio 30 ms (wild type 1.04 ± 0.03 ms, *n* = 16; *Myo15*^ΔMyTH4 (1+2)^ 0.97 ± 0.01, *n* = 15). **(c)** Confocal images of *myo15*^*−/−*^ mutant and control NMJs treated with PhTx (fire LUT), scale bar 2 µm. **(d)** Quantification of c. BRP mean pixel intensity (wild type - PhTx 100.0 ± 7.59%, *n* = 11; + PhTx 203.2 ± 17.60%, *n* = 12; *Myo15*^*−/−*^ mutant - PhTx 100.0 ± 10.35%, *n* = 11; + PhTx 111.8 ± 17.76%, *n* = 8. **(e)** mEPSP traces of Myo15 knock-down and control NMJs with (red) and without (black) PhTx treatment for 10 min, scale bar 5 mV, 500 ms. **(f)** mEPSP traces of Myo15 knock-down and control NMJs with (red) and without (black) PhTx treatment for 30 min, scale bar 2 mV, 500 ms. **(g)** Quantification of MTM 1 h of Gal80^ts^;ok107-Gal4>Myo15-RNAi^101729^ without induction (restrictive 18°C temperature, control driver line PI 0.39 ± 0.061, Myo15 knock-down (RNAi^101729^) PI 0.50 ± 0.054, control RNAi PI 0.43 ± 0.052, *F*_(*2*,*23*)_ = 0.9187, P = 0.4145, *n* = 8). **(h)** Flies expressing RNAi-Myo15^101729^ in the adult MB lobes present normal olfactory acuity to octanol (control driver line OA 0.52 ± 0.060, Myo15 knock-down (RNAi^101729^) OA 0.383 ± 0.0598, control RNAi OA 0.542 ± 0.085; *F*_(*2*,*17*)_ = 1.530, P = 0.2486, *n* = 6) and methylcyclohexanol (control driver line OA 0.55 ± 0.0629, Myo15 knock-down (RNAi^101729^) OA 0.728 ± 0.0682, control RNAi OA 0.740 ± 0.0879; *F*_(*2*,*17*)_ = 2.046, P = 0.1639, *n* = 6). **(i)** Those flies present normal electrical shock reactivity (control driver line reactivity 0.79 ± 0.045, Myo15 knock-down (RNAi^101729^) reactivity 0.855 ± 0.039, control RNAi reactivity 0.868 ± 0.0566; *F*_(*2*,*17*)_ = 0.7134, P = 0.5059, *n* = 6). **(j)** Quantification of MTM 1 h for flies expressing RNAi-Myo15^104606^ in the adult MB lobes (control driver line PI 0.21 ± 0.016, Myo15 knock-down (RNAi^104606^) PI 0.05 ± 0.054, control RNAi PI 0.29 ± 0.046; *F*_(*2*,*23*)_ = 8.370, P = 0.0021, *n* = 8, post hoc Tukey’s multiple comparisons test, Gal80^ts^;ok107-Gal4/+ versus Gal80^ts^;ok107-Gal4/RNAi^104604^, *P < 0.05, +/RNAi^104604^ versus Gal80^ts^;ok107-Gal4/RNAi^104604^, **P < 0.01). **(k)** Quantification of STM for those flies (control driver line PI 0.18 ± 0.008, Myo15 knock-down (RNAi^104606^) PI 0.18 ± 0.047, control RNAi PI 0.25 ± 0.028; *F*_(*2*,*17*)_ = 1.414, P = 0.2738, *n* = 6). **(l)** Flies expressing RNAi-Myo15^104606^ in the adult MB lobes present normal olfactory acuity (OA) to octanol (control driver line OA 0.56 ± 0.083, Myo15 knock-down (RNAi^104606^) OA 0.403 ± 0.0577, control RNAi OA 0.477 ± 0.065; *F*_(*2*,*17*)_ = 1.333, P = 0.2933, *n* = 6) and methylcyclohexanol (control driver line OA 0.60 ± 0.0733, Myo15 knock-down (RNAi^104606^) OA 0.713 ± 0.107, control RNAi OA 0.642 ± 0.093; *F*_(*2*,*17*)_ = 0.3771, P = 0.6922, *n* = 6). **(m)** Those flies present normal electrical shock reactivity (control driver line reactivity 0.87 ± 0.029, Myo15 knock-down (RNAi^104606^) reactivity 0.865 ± 0.0425, control RNAi reactivity 0.843 ± 0.0189; *F*_(*2*,*17*)_ = 0.2614, P = 0.7734, *n* = 6). All data are provided as mean ± SEM. **(A–b and d–f)***n* = NMJs with 1–2 NMJs/animal, four to six animals, g–m: n = cohort of ∼50 adult flies, 5–8 cohorts measured. ****P < 0.0001; ***P < 0.001; **P < 0.01; *P < 0.05; n.s., not significant. PI, performance index.

Myo15 depletion resulted in smaller AZs and an increase in AZ density. To determine whether this AZ remodeling affected baseline synaptic transmission and physiological properties of the synaptic terminal, we performed two-electrode voltage-clamp (TEVC) recordings in Myo15-RNAi knock-down and *Myo15*^ΔMyTH4 (1+2)^ deletion mutants. While evoked release amplitudes remained unchanged (Fig. S5, A, B, O, and P), release kinetics were significantly accelerated, with faster rise times and decay τ constants (Fig. S5, C, D, Q, and R), though evoked charge was unaffected (Fig. S5, E and S) in both genetic conditions. For spontaneous release, amplitudes remained unchanged (Fig. S5, H, I, V, and W), but kinetics were again accelerated (Fig. S5, J, K, X, and Y), while frequency remained stable (Fig. S5, L and Z). Since the spontaneous and evoked release kinetics were accelerated both in the presynaptic knock-down and mutant background, these deficits might arise from changes in the response kinetics of postsynaptic glutamate receptors as a global compensatory plasticity mechanism in response the presynaptic alterations (Goel and Dickman, 2018; Goel et al., 2019; Goel et al., 2020). Paired-pulse analysis at 10 and 30 ms (Fig. S5, F, G, M, N, T, U, a, and b) revealed no abnormalities, suggesting that SV release machinery functionality was not fundamentally altered in the absence of Myo15 function.

**Figure 7.**
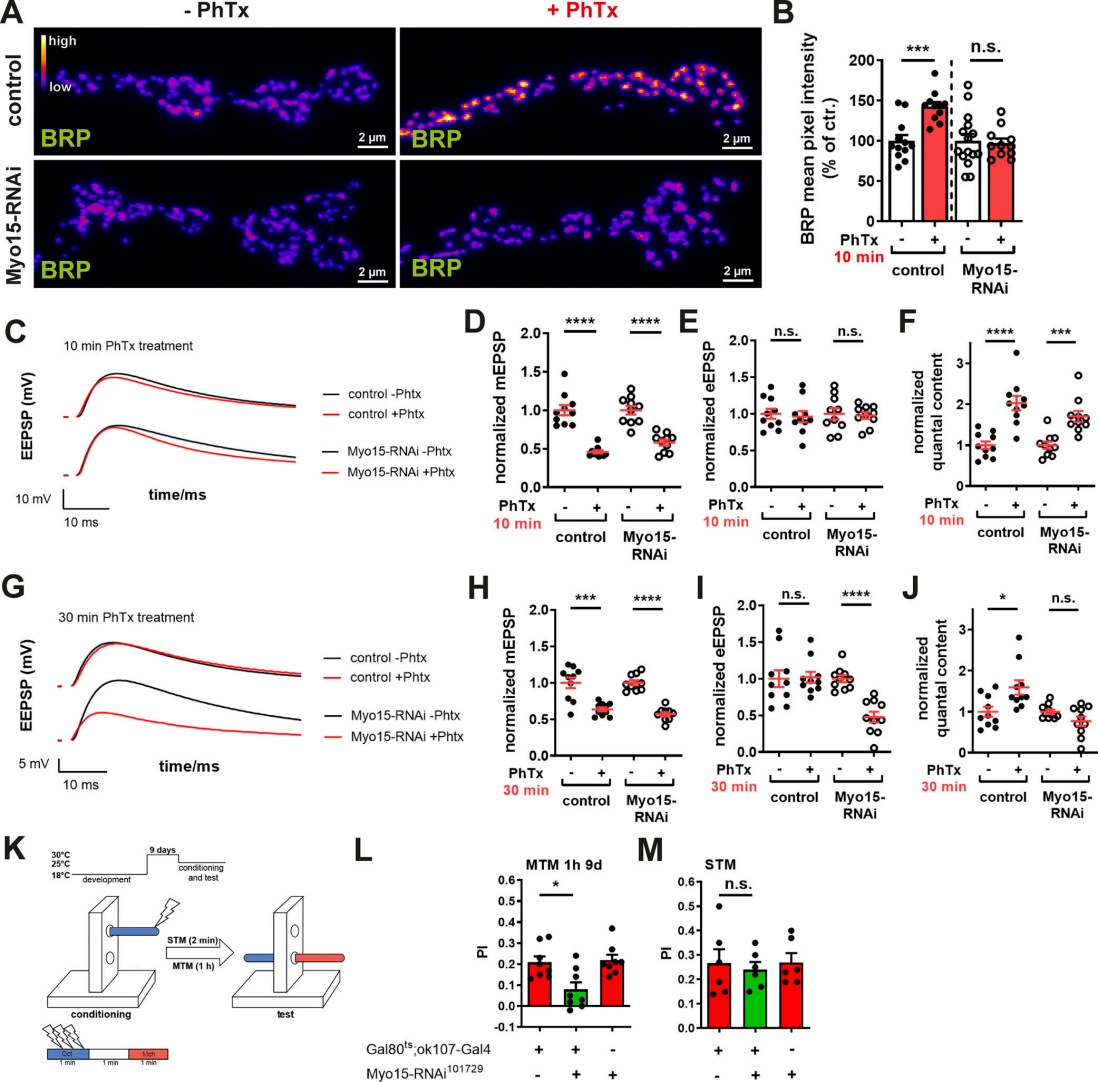
**PHP and sustained memory consolidation depend on Myo15. (A)** Confocal images of Myo15-RNAi–mediated knock-down and control NMJs treated with PhTx to induce PHP stained for BRP (fire LUT), scale bar 2 µm. **(B)** Quantification of A, BRP mean pixel intensity (control - PhTx 100.0 ± 7.21%, *n* = 12; + PhTx 142.5 ± 5.67%, *n* = 11; Myo15-RNAi - PhTx 100.0 ± 8.54%, *n* = 15; + PhTx 97.16 ± 5.65%, *n* = 11). **(C)** Representative traces of evoked EPSPs after 10 min PhTx treatment at control (black) and Myo15-RNAi knock-down (red) third-instar muscle 6/7 NMJs, scale bars 10 ms, 10 mV. **(D and E)** Quantification of 10 min PhTx treatment, *n* = 10. **(D)** Normalized mEPSPs (control - PhTx 1.00 ± 0.066%; + PhTx 0.46 ± 0.021%; Myo15-RNAi - PhTx 1.00 ± 0.056%; + PhTx 0.58 ± 0.038%). **(E)** Normalized eEPSPs (control - PhTx 1.00 ± 0.067%; + PhTx 0.96 ± 0.076%; Myo15-RNAi - PhTx 1.00 ± 0.077%; + PhTx 0.98 ± 0.045%). **(F)** Normalized quantal content (control - PhTx 1.00 ± 0.096%; + PhTx 2.03 ± 0.176%; Myo15-RNAi - PhTx 1.00 ± 0.088%; + PhTx 1.71 ± 0.134%). **(G)** Representative traces of evoked EPSPs after 30 min PhTx treatment at control (black) and Myo15-RNAi knock-down (red) third-instar muscle 6/7 NMJs, scale bars 10 ms, 5 mV. **(H–J)** Quantification of 30 min PhTx treatment, *n* = 10. **(H)** Normalized mEPSPs (control - PhTx 1.00 ± 0.072%; + PhTx 0.636 ± 0.028%; Myo15-RNAi - PhTx 1.00 ± 0.034%; + PhTx 0.57 ± 0.031%). **(I)** Normalized eEPSPs (control - PhTx 1.00 ± 0.114%; + PhTx 1.02 ± 0.076%; Myo15-RNAi - PhTx 1.00 ± 0.048%; + PhTx 0.48 ± 0.073%). **(J)** Normalized quantal content (control - PhTx 1.00 ± 0.121%; + PhTx 1.59 ± 0.176%; Myo15-RNAi - PhTx 1.00 ± 0.055%; + PhTx 0.77 ± 0.115%). **(K)** Schematic representation of aversive olfactory learning setup. **(L)** Quantification of MTM 1 h (control driver line PI 0.21 ± 0.028, Myo15 knock-down (RNAi^101729^) PI 0.08 ± 0.033, control RNAi PI 0.22 ± 0.026, *F*_(*2*,*23*)_ = 7.056, P = 0.0045, *n* = 8, post hoc Tukey’s multiple comparisons test, Gal80^ts^;ok107-Gal4/+ versus Gal80^ts^;ok107-Gal4/RNAi^104604^, *P < 0.05, +/RNAi^104604^ versus Gal80^ts^;ok107-Gal4/RNAi^104604^, **P < 0.01). **(M)** Quantification of STM (control driver line PI 0.27 ± 0.057, Myo15 knock-down (RNAi^101729^) PI 0.24 ± 0.031, control RNAi PI 0.27 ± 0.038, *F*_(*2*,*17*)_ = 0.1446, P = 0.8666, *n* = 6). All data are provided as mean ± SEM. **(B–J)***n* = NMJs with 1–2 NMJs/animal, four to six animals, L and M: n = cohort of ∼50 adult flies, 6–8 cohorts measured. ****P < 0.0001; ***P < 0.001; **P < 0.01; *P < 0.05; n.s., not significant. PI, performance index.

Opposed to chronic loss of Myo15 function, we asked next if Myo15 might play a role in an acute AZ remodeling scenario, testable by PHP paradigms. PHP is a compensatory increase in presynaptic release induced here by partial blockade of postsynaptic ionotropic glutamate receptors using philanthotoxin (PhTx) (Frank et al., 2006; Davis and Muller, 2015; Nair et al., 2021). A hallmark of PHP is rapid AZ scaffold remodeling within minutes to counteract reduced postsynaptic sensitivity (Bohme et al., 2019; Mrestani et al., 2021). In agreement with previous findings (Weyhersmuller et al., 2011; Goel et al., 2017; Bohme et al., 2019), 10 min of PhTx application increased BRP mean pixel intensity by 50% (Fig. 7, A and B). However, Myo15 depletion abolished this response, both under RNAi-mediated knock-down and in *myo15*^*−/−*^ mutants (Fig. 7, A and B; and Fig. S5, c and d). Since BRP loss affects PHP only at 30 min of PhTx treatment (sustained phase) but not at 10 min (acute phase) (Turrel et al., 2022), we investigated whether Myo15 was required for both phases. Successful PhTx-mediated glutamate receptor blockade at 10 and 30 min was confirmed by reduced spontaneous miniature EPSP (mEPSP) amplitudes (Fig. 7, C, D, G, and H; and Fig. S5, E and F). At 10 min, acute PHP was still intact in Myo15-depleted terminals, as eEPSP amplitudes were restored by increased presynaptic neurotransmitter release (Fig. 7, C, E, and F). However, at 30 min, PHP could not be maintained, mirroring the phenotype observed in BRP mutants (Turrel et al., 2022; Ramesh et al., 2023). Here, eEPSP amplitudes were reduced by 50%, and quantal content could not be increased (Fig. 7, G, I, and J). These findings demonstrate that Myo15 is required for presynaptic AZ remodeling during sustained PHP, suggesting a crucial role in long-term synaptic homeostasis.

### Memory consolidation by mushroom body intrinsic neurons is Myo15 dependent

Synaptic plasticity is a fundamental mechanism underlying memory formation and storage in the brain (Marder and Goaillard, 2006; Turrigiano, 2012; Takeuchi et al., 2014). PHP, a conserved mechanism that stabilizes synaptic transmission, was recently linked to aversive olfactory memory formation in the *Drosophila* mushroom body (MB) (Davis and Muller, 2015; Turrel et al., 2022). We recently provided evidence that postdevelopmental BRP knock-down within neurons of the MB severely hampered olfactory aversive mid-term memory (MTM) measured a few hours after conditioning, while this constellation still allowed for appropriate learning in the range of minutes (short-term memory, STM) (Turrel et al., 2022). Given Myo15’s role in sustained plasticity at motoneuronal synapses, we hypothesized that it might be required for plasticity-based olfactory memory formation in adult fly brains. To test this, we used RNAi-mediated Myo15 knock-down in MB Kenyon cells (KCs) via the TARGET system (McGuire et al., 2003). Gal80ts was used to restrict RNAi expression during development at 18°C, followed by postdevelopmental Myo15 depletion for 9 days at 29°C. Flies were tested using aversive olfactory conditioning, with STM determined immediately and MTM at 1 h (Fig. 7 K). Postdevelopmental Myo15 depletion in MB neurons significantly impaired MTM (Fig. 7 L), but not STM (Fig. 7 M). Memory defects were not observed at the restrictive temperature when RNAi expression was suppressed (Fig. S5 g). The intact STM formation (Fig. 7 M) suggests that Myo15 depletion does not impair overall KC function, and Myo15-depleted neurons can still appropriately sustain odor and electric shock association (Fig. S5, h and i), indicating that MTM deficits are specific. These results—impaired MTM with preserved STM—mirror previously reported memory deficits following BRP and transport protein knock-down (kinesin Kif1α/Unc104 [Turrel et al., 2022] and its adaptor Arl8 [Vukoja et al., 2018]) using a comparable inducible 9-day postdevelopmental depletion paradigm, where overall neuronal structure was not affected (Turrel et al., 2022). Myo15 memory impairment was confirmed by a second, independent RNAi line, showing a specific MTM deficit (Fig. S5 j), while STM was not affected (Fig. S5 k), implying functionality of KCs able to properly sustain odor (Fig. S5 l) and electric shock association (Fig. S5 m) and excluding RNAi-induced off-target effects. Our findings propose Myo15 as an important molecular regulator of memory storage mechanisms.

## Discussion

Presynapses, composed of the AZ scaffold and associated SVs, are highly specialized subcellular compartments that orchestrate neurotransmitter release. This cytoplasmic scaffold ensures stable yet adaptive neurotransmission, which underlies fundamental brain functions, including learning and memory (Sudhof, 2004; Sudhof, 2012; Petzoldt et al., 2016; Emperador-Melero and Kaeser, 2020). While actin requirement and function in the context of SV release have been extensively studied (Dillon and Goda, 2005; Cingolani and Goda, 2008; Rust and Maritzen, 2015; Soykan et al., 2017; Gentile et al., 2022; Wu and Chan, 2022), the interplay between actin or MT dynamics and AZ assembly, maintenance, and plastic remodeling remains less understood. Actin remodeling factors such as spinophilin (Spn/NAB1) are reported to recruit AZ-adjacent F-actin to facilitate biosynthetic cargo incorporation (Chia et al., 2012; Ramesh et al., 2023). MTs, while primarily linked to synaptic terminal outgrowth and stabilization (Roos et al., 2000; Pielage et al., 2008), have also been implicated in local AZ organization, as suggested by the roles of the MAP1 homolog futsch in AZ stabilization (Lepicard et al., 2014) and the formin DAAM1 in MT organization near AZs (Migh et al., 2018). Given that AZ assembly and remodeling relies on biosynthetic cargo trafficking and cytoskeletal dynamics (Chia et al., 2012; Kern et al., 2013; Lepicard et al., 2014; Niwa et al., 2017; Vukoja et al., 2018; Ramesh et al., 2023), an in-depth understanding of how these processes are coordinated is essential.

Here, we identify the unconventional class XV myosin Myo15 as a novel regulator of AZ assembly and remodeling. Myo15, also referred to as Sisyphus/Myo10A (Tzolovsky et al., 2002; Liu et al., 2008; Rich et al., 2021), is the largest *Drosophila* myosin and possesses a conserved tail region containing MyTH4, FERM-like, and MyTH4-FERM domains, where the FERM domain mediates cargo binding and the MyTH4 domain has been implicated in MT binding (Liang et al., 1999; Weber et al., 2004; Hirano et al., 2011; Lu et al., 2014; Planelles-Herrero et al., 2016; Rich et al., 2021). Our data show that Myo15 regulates AZ scaffold size in a dose-dependent manner: Myo15 depletion reduced AZ size while increasing AZ density, whereas Myo15 overexpression enlarged individual AZs and extended NMJ terminal length. Myo15 localizes to presynaptic boutons, where it partially overlaps with the actin meshwork and MTs. However, it does not appear to be a core AZ component, but rather resides close and adjacent to a subset of AZ scaffolds, while AZ size is not related to either the amount or the distance of AZs close to Myo15 clusters.

### Myo15 regulates the presynaptic actin meshwork, NMJ morphology, and AZ size

Myo15 overexpression led to a denser actin network and enlarged actin clusters, suggesting an actin-stabilizing function, whereas Myo15 depletion caused an increase in actin clusters and an accumulation of actin in additionally formed satellite boutons. These findings align with studies on the WAVE complex component Cyfip, whose disruption similarly alters actin organization and NMJ morphology, leading to expanded inter-bouton regions and satellite bouton formation (Zhao et al., 2013). Furthermore, Myo15 was previously shown to both spatiotemporally control actin stability by positioning of MICAL, a flavoprotein monooxygenase that destabilizes actin, and to rebuild local cytoskeleton in the disassembled areas through its cargo-trafficking function (Rich et al., 2021). Consequently, it is conceivable that Myo15 promotes local actin formation through the localization of actin regulators, such as formins, e.g., DAAM shown to couple the AZ scaffold to the presynaptic cytoskeleton (Migh et al., 2018), or Cyfip (Zhao et al., 2013), a task for future investigation. Given these parallels, Myo15 possibly regulates local actin organization, potentially influencing presynaptic material or organelle positioning and thereby influencing NMJ morphology and presynaptic cargo dynamics to affect AZ assembly across the synaptic terminal.

Next to actin, Myo15 decorates long-range MTs at synaptic terminals and other tissues, displaying a partially punctate localization pattern. Our deletion analysis of the MyTH4 domains, previously shown to mediate MT binding (Weber et al., 2004; Hirano et al., 2011), indicates that these domains are essential for Myo15 function. However, STED imaging did not reveal significant alterations in Myo15-MT distance upon MyTH4 domain deletion, suggesting that Myo15 positioning is further determined by parallel interactions with actin. Previous studies in *Drosophila* embryos have shown that the Myo15 tail interacts with α-tubulin, EB1, and aPKC and balances actin–MT interactions at filopodia tips during closure (Liu et al., 2008), raising the possibility of a broader cytoskeletal coordination function by affecting MT assembly at the synaptic terminal. Of note, Myo15 localization to MTs could also result from trafficking transport vesicles with a myosin cargo. It is further tempting to speculate that Myo15, as the largest *Drosophila* myosin with splice variants up to 3145-aa long, might link actin and MTs at assembling and remodeling AZs to locally regulate cargo supply, possibly in conjunction with futsch, the *Drosophila* MAP1 homolog (Hummel et al., 2000), shown to bridge MT and the distal end of AZs (Lepicard et al., 2014). However, the precise mechanism underlying Myo15’s interaction with the cytoskeleton remains to be elucidated in future studies.

Interestingly, phenotypes resembling Myo15 depletion, including AZ reduction, increased synapse number, and satellite bouton formation, have been reported for other endocytosis mutants, including endophilin (Dickman et al., 2006; Goel et al., 2019) or nervous wreck in conjunction with Dap160/intersectin (O’Connor-Giles et al., 2008). As Myo15 also affects EE architecture as well as SV amount or distribution, and given that endophilin regulates AZ size through vesicle recycling or distribution of AZ components from a local supply pool (Bohme et al., 2019), it is plausible that Myo15 might influence AZ structure via related mechanisms. Interestingly, previous studies suggest that global, NMJ-wide plasticity mechanisms ensure the maintenance of total synaptic strength upon altered presynaptic cargo supply in the context of endophilin mutants (Goel et al., 2019). The same principle could apply to the observed Myo15 knock-down phenotypes, as it appears that global neurotransmission of a given synaptic terminal upon chronic Myo15 loss-of-function remained stable, likely due to the decrease in individual AZ size being balanced by an increase in AZ number, maintaining the total number of SV release sites and total transmission.

### Myo15-dependent AZ remodeling sustains homeostatic plasticity and olfactory memory

Myo15 depletion impaired sustained PHP, tested in a PhTx paradigm-blocking postsynaptic glutamate receptors for 30 min, while leaving the acute phase intact (10 min PhTx application). PHP, which compensates for reduced postsynaptic receptor function, requires structural AZ remodeling, particularly during its sustained phase (Frank et al., 2006; Davis and Muller, 2015, Nair et al., 2021; Turrel et al., 2022; Ramesh et al., 2023). Notably, Myo15 was previously shown to spatiotemporally position the actin disassembling regulator MICAL (Rich et al., 2021), thus possibly locally destabilizing cortical AZ close actin and, likely as a direct consequence, facilitating SV fusion during PHP (Terman et al., 2002; Orr et al., 2017; Orr et al., 2022; Ramesh et al., 2023). Therefore, Myo15 may regulate PHP by positioning MICAL at AZs to facilitate actin disassembly and SV release. Furthermore, Myo15 was found to interact with spinophilin/NAB1 (Ramesh et al., 2023), which modulates actin dynamics at AZs and could serve as an intermediary protein in this process.

Beyond synaptic plasticity, we found that Myo15 plays an important role in memory consolidation. Learning and memory require synaptic modifications, and previous work has demonstrated that AZ remodeling supports MTM consolidation in *Drosophila* MB neurons (Turrel et al., 2022). Our findings show that Myo15 depletion selectively impairs mid-term, but not short-term, olfactory memory. Given its role in AZ remodeling and sustained PHP, Myo15 likely contributes to memory consolidation by ensuring stable synaptic plasticity over timescales of minutes to hours.

### Evolutionary perspectives on Myo15 and vertebrate Myo10

Myo15 is the only class XV myosin in *Drosophila* and is homologous to vertebrate Myo15A (Tzolovsky et al., 2002; Liu et al., 2008). Mutations in human and mouse Myo15A cause DFNB3-associated deafness and vestibular dysfunction (Probst et al., 1998; Wang et al., 1998; Anderson et al., 2000; Rehman et al., 2016), with defects in stereocilia growth and actin bundle regulation (Belyantseva et al., 2005; Delprat et al., 2005; Fang et al., 2015; Rehman et al., 2016). Myosins XV, VII, and X share a phylogenetic branch and carry MyTH4-FERM domains, suggesting functional similarities (Sellers, 2000; Odronitz and Kollmar, 2007). Unlike vertebrates, which retain all three classes, *Drosophila* lost Myo10, raising the possibility that *Drosophila* Myo15 compensates for both vertebrate Myo15 and Myo10 functions. Interestingly, vertebrate Myo10, which is enriched in the brain, is essential for neural tube closure and axonal pathfinding (Berg et al., 2000; Sousa et al., 2006). Similar to Myo15, it modulates actin dynamics by clustering filaments at filopodia tips via Ena/VASP transport (Tokuo and Ikebe, 2004) and stabilizing actin filaments during filopodia extension (Berg and Cheney, 2002). Additionally, Myo10 interacts with MTs at the meiotic spindle (Weber et al., 2004), reinforcing the idea that Myo15 and Myo10 share a conserved function in coordinating cytoskeletal elements. Future studies could explore whether Myo15 regulates actin and MT organization through similar mechanisms in synapses.

### Conclusion

Our study identifies Myo15 as a novel regulator of AZ assembly and plastic remodeling in *Drosophila*. Likely by modulating actin and possibly MT stability, Myo15 regulates AZ size and density, SV distribution, and potentially endosomal organization. Its function is crucial for sustained homeostatic plasticity and memory consolidation, highlighting its role in synaptic stability and adaptability. Given its evolutionary conservation and parallels with vertebrate Myo10, Myo15 provides a valuable model for studying cytoskeletal coordination in synaptic function. Future work should dissect its precise molecular interactions and regulatory mechanisms at the synapse.

## Materials and methods

### Fly husbandry

*Drosophila melanogaster* strains were reared under standard laboratory conditions and raised at 25°C and 70% humidity on semi-defined medium (Bloomington recipe). For RNAi and overexpression experiments, flies were kept at 29°C. For electrophysiological recordings, only male larvae were used. For all other experiments, both male and female animals were used, except for rescue, where only male larvae were used. For genotypes and fly strains used, see Tables 2 and 3.

**Table 2.** Fly genotypes and strains

Fly strains	Source	Identifier
w^1118^ as wild type	Bloomington *Drosophila* Stock Center (BDSC)	#3605
ok6-Gal4/II	BDSC	#64199
elaV-Gal4/X	BDSC	#458
ok107-Gal4/IV	Aso et al. (2009)	
UAS-dicer2/II	Vienna *Drosophila* Resource Center (VDRC)	#60008
Myo15 gRNA/II	This study	
UAS-Cas9/III	BDSC	#67085
UAS-Myo15-RNAi/II	VDRC	#101729
UAS-Myo15-RNAi/III	VDRC	#33486
UAS-Myo15-RNAi/II	VDRC	#104604
UAS-Myo15-GFP/II or III	BDSC	#24781/2
UAS-Myo15-RFP/II or III	BDSC	#24784/5
UAS-Myo15-RFP (Syph-RFP) UAS-Myo15-GFP (Syph-GFP)	Gift from Terman lab, UT Southwestern, Dallas, TX, USA (Rich et al., 2021)	
UAS-actin5C-GFP/III	BDSC	#9257
*myo15-/myo15-* on X	This study	
Myo15ΔMyTH4 (1)/X	This study	
Myo15ΔMyTH4 (1+2)/X	This study	
Myo15ΔFERM/X	This study	
Tubulin-Gal80^ts^/II	BDSC	#7019
RNAi lines from myosin screen		
CG 2146 (didum)-RNAi/III	VDRC	#44292
CG 5125 (ninaC)-RNAi/III	BDSC	#27693
CG 5501 (Myo 95E)-RNAi/III	VDRC	#51207
CG 5695 (jaguar)-RNAi/III	BDSC	# 28064
CG 6976 (Myo 28B1)-RNAi/II	VDRC	# 101016
CG 7438 (Myo ID)-RNAi/III	BDSC	# 33971
CG 7595 (crinkled)-RNAi/III	VDRC	# 9265
CG 9155 (Myo IC)-RNAi/II	VDRC	# 110682
CG 10595 (dachs)-RNAi/II	VDRC	# 102550
CG 15792 (zipper)-RNAI/III	BDSC	# 36727
CG 17927 (mhc)-RNAi/III	BDSC	# 35729

**Table 3.** Crosses and genotypes used

Crosses	Experiment
ok6-Gal4, UAS-dicer2 x UAS-Myo15-RNAi^101729^; UAS-Myo15-RNAi^33486^, control: ok6-Gal4, UAS-dicer2 x w1118	RNAi-mediated knock-down
ok6-Gal4; UAS-Cas9 x Myo15 gRNA, control: ok6-Gal4; UAS-Cas9 x w1118	gRNA-mediated knock-down
ok6-Gal4 x UAS-Myo15-GFP/RFP, control: ok6-Gal4 x w1118	Overexpression (NMJ and salivary gland)
Mutant: *myo15-*/*myo15-*control: Wild type (w1118), elaV-Gal4 x UAS-Myo15-GFP, control: elaV-Gal4 x w1118	Western blots (larva), Western blots (adult brain)
ok6-Gal4 x UAS-actinGFP, ok6-Gal4, UAS-actinGFP × UAS-Myo15RFP, ok6-Gal4, UAS-actinGFP × UAS-Myo15-RNAi^101729^; UAS-Myo15-RNAi^33486^	Actin and Myo15 overexpression
ok6-Gal4; UAS-Myo15-RFP ×, UAS-Myo15-RNAi^101729^; UAS-Myo15-RNAi^33486^ ok6-Gal4; UAS-Myo15-RFP × w1118, control: ok6-Gal4 × w1118	Myo15 overexpression and RNAi simultaneously
Rescue: *myo15-*/FM7i, act-GFP; ok6-Gal4/II x *myo15-*/Y; UAS-Myo15^GFP^/II, Mutant: *myo15-*/*myo15-*control: Wild type (w1118)	Presynaptic rescue
Tubulin-Gal80^ts^; ok107-Gal4 x UAS-Myo15-RNAi^101729^; UAS-Myo15-RNAi^33486^ or UAS-Myo15-RNAi^104604^	Conditional expression of RNAi in MB

### Generation of fly lines

#### Myo15gRNA

The Myo15gRNA strain was generated by Well Genetics, Inc. In short, a Myo15/CG43657 gRNA (CRISPR target site [PAM]: 5′-CTG​CTG​GAC​GAT​CTG​CAC​G[AGG]-3′) was generated by gene synthesis (gRNA primers: Sense oligo 5′-GTC​GCC​TGC​TGG​ACG​ATC​TGC​ACG-3′; Antisense oligo 5′-AAA​CCG​TGC​AGA​TCG​TCC​AGC​AGG-3′). The gRNA would target the large major Myo15 isoforms (RF, RC, and RD) but not the short Myo15-RE isoform. The gRNA was cloned into the BbsI sites of the pCFD3-dU6:3gRNA vector using the restriction cloning method (digested by BbsI [Thermo Fisher Scientific], ligated with annealed gRNA, followed by standard transformation protocol, colony PCR selection, and sequencing). The pCFD3-Myo15 plasmid was used for microinjection into the fly strain [LWG126] y[1] M[vas-int.Dm]ZH-2A w[*]; P[y[+t7.7] = CaryP]attP40 (landing site attp40). The gRNA is expressed ubiquitously in all cells due to the U6 promoter of pCFD3 and crossed to ok6-Gal4; UAS-Cas9 to specifically guide Cas9 to the Myo15 locus in motoneurons.

#### Myo15 domain deletions: Myo15^ΔMyTH4 (1+2)^, Myo15^ΔMyTH4 (1)^, and Myo15^ΔFERM^

The domain deletions were generated by Well Genetics, Inc., based on the modified methods by Kondo and Ueda (2013) using the CRISPR-Cas9–mediated genome-editing approach by homology-dependent repair using one gRNA and a double-stranded DNA plasmid donor. For all three constructs (primers below), in brief, the gRNA sequence 5′-GGC​CGA​CTA​TAT​TGT​GCA​CA[AGG]-3′/5′-GGC​ACT​ACC​GCT​AAC​TCT​GC[CGG]-3′ was cloned into the U6 promoter plasmid. Cassette PBacDsRed containing two PBac terminals and 3xP3-DsRed and two homology arms was cloned into pUC57-Kan as a donor template for repair. *Myo15/CG43657*-targeting gRNAs, and hs-Cas9 were supplied in DNA plasmids, together with a donor plasmid for microinjection into embryos of the control strain *w[1118]*. F1 flies carrying the selection marker of 3xP3-DsRed were further validated by genomic PCR and sequencing. CRISPR generated a 111 aa deletion in the MyTH4 (1) domain (RD-isoform 1061–1171 aa, leaving the MyTh4 (1) domain 47 aa before and 2 aa after the deletion undeleted) of Myo15. The MyTH4 (1) deletion mutant was subsequently used to excise the MyTH4(2) domain (as described above). CRISPR generated a 109 aa deletion in the MyTH4 (2) domain (RD-isoform 2702–2810 aa, leaving the MyTh4 (2) domain 44 aa before and 2 aa after the deletion undeleted) of Myo15. CRISPR generated a 115 aa deletion in the FERM domain (RD-isoform 2919–3033 aa, leaving the FERM domain 105 aa before the deletion undeleted) of Myo15. All deletion regions were replaced by cassette PBacDsRed. Last, the PBacDsRed was removed by FRT-mediated excision (using # 8285; BDSC, *w*^1118^; *CyO*, *P[Tub-PBac\T]2/wgSp-1*) to generate the final domain deletion lines, lines verified by PCR as follows: genomic DNA was obtained from a single fly of each stock following single-fly DNA prep. Injection strain *w1118* was used as a negative control. PCR was performed using KOD-FX (TOYOBO) on BioRad S1000 Thermal Cycler.1 (primer indicated below). Final validation of excision was carried out by genomic sequencing.

Primers are as follows for the different constructs:

#### Myo15^ΔMyTH4 (1)^

gRNA1 (upstream)

Cutting site: between X: 10944522–10944523

gRNA primers:

Sense oligo 5′-CTT​CGG​CCG​ACT​ATA​TTG​TGC​ACA-3′
Antisense oligo 5′-AAA​CTG​TGC​ACA​ATA​TAG​TCG​GCC-3′
Upstream homology arm: 1,065 bp, X: 10943439-1094450
Forward oligo: 5′-CCA​ACG​GTA​GCA​GCC​AGA​TT-3′
Reverse oligo: 5′-TAG​CTT​TTC​CTT​GGC​GCC​TT-3′

gRNA2 (downstream)

Cutting site: between X: 10944873–10944874

gRNA primers:

Sense oligo 5′-CTT​CGG​CAC​TAC​CGC​TAA​CTC​TGC-3′
Antisense oligo 5′-AAA​CGC​AGA​GTT​AGC​GGT​AGT​GCC-3′
Downstream homology arm: 996 bp, X: 10944871–10945866
Forward oligo: 5′-CTC​TGC​CGG​ACG​AGG​CCA-3′
Reverse oligo: 5′-CCA​AGT​TCT​GGA​CGA​GTG​GT-3′

PCR primer for validation:

OWG8778 5′-TGA​TCG​TCA​TCT​GGT​CAA​GG-3′
OWG8779 5′-CAG​CTC​CTT​CTC​TGC​AAT​CA-3′

#### Myo15^ΔMyTH4 (1+2)^

gRNA1(upstream)

Cutting site between X: 10961477–10961478

gRNA primers:

Sense oligo 5′-CTT​CGC​ACT​TGA​CCT​CGG​TGA​GGT​C-3′
Antisense oligo 5′-AAA​CGA​CCT​CAC​CGA​GGT​CAA​GTG​C-3′
Upstream homology arm: 1,081 bp, X: 10960386–10961466
Forward oligo: 5′-CCT​GGT​CTA​AAA​CAC​CTT​GAT​GA-3′
Reverse oligo: 5′-GTC​GGG​ATC​CAG​CGG​TAT​ATC​A-3′

gRNA2 (downstream)

Cutting site between X: 10962400–10962401

gRNA primers:

Sense oligo 5′-CTT​CGA​GGT​GAC​GGC​CGT​GTC​CGC-3′
Antisense oligo 5′-AAA​CGC​GGA​CAC​GGC​CGT​CAC​CTC-3′
Downstream homology arm: 996 bp, X: 10962396–10963383
Forward oligo: 5′-CCG​AGG​TCG​TGT​CCG​CTG​GAC​GTT​CA-3′
Reverse oligo: 5′-CTC​CGT​TTG​GAC​ACG​GAT​CA-3′

PCR primer for validation:

OWG9913 5′-ACG​ACC​TGG​ATG​CCA​ACT​AT-3′
OWG9916 5′-AGC​AGC​AGA​CCC​TCC​AGA​TA-3′

#### Myo15^ΔFERM^

gRNA1 (upstream)

Cutting site between X: 10962716–10962717

gRNA primers:

Sense oligo 5′-CTT​CGA​CCT​CCA​CGT​AGA​GCG​GCA​T-3′
Antisense oligo 5′-AAA​CAT​GCC​GCT​CTA​CGT​GGA​GGT​C-3′
Upstream homology arm: 1,041 bp, X: 10961679–10962719
Forward oligo 5′-ACT​TAA​GCA​CCC​GAA​CCG​GA-3′
Reverse oligo 5′-CGG​CAT​GGG​AGC​CGG​TTC​T-3′

gRNA2 (downstream)

Cutting site between X: 10963041–10963042

gRNA primers:

Sense oligo 5′-CTT​CGC​TGC​TGC​CGA​AGA​GCG​GCC-3′
Antisense oligo 5′-AAA​CGG​CCG​CTC​TTC​GGC​AGC​AGC-3′
Downstream homology arm: 1,085 bp, X: 10963073–10964157
Forward oligo 5′-GCG​CAT​TTG​GGC​GGA​GGA-3′
Reverse oligo 5′-TTG​TTC​AGT​TAA​GGG​CCC​AC-3′

PCR primer for validation:

OWG8780 5′-CTG​ATG​TCA​TTG​CCG​AAC​TG-3′
OWG8781 5′-CAG​ATT​GCC​CAC​CTT​CAT​GT-3′

#### Myo15^−/−^ mutant flies

The Myo15-specific chromosomal deficiency was created by a flippase-mediated excision of the Myo15-coding region between two P-element insertions according to Thibault et al. (2004); Bellen et al. (2011). Both PBac[wh] transposons (integration sites: PBac[WH]f06507 [FBti0052988] X:10,935,969.10,935,969 and PBac[WH]f03968 [FBal0160159] X:10,963,972.10,963,972) contained FRT sites in a distal orientation, allowing for excision. Females of the following genotype were crossed: PBac[WH]f06507/PBac[WH]f03968; hs-Flp. After heat shock, progenies were screened for the absence of [w^+^] and sequenced to verify the Myo15 deletion. For details and resources, contact Jörg Grosshans.

### Immunostainings of larval NMJs

#### Immunohistochemistry for confocal and STED microscopy

For immunohistochemistry, dissections were performed in haemolymph-like solution 3 (HL3 [Stewart et al., 1994]; composition in mM: 70 NaCl, 5 KCl, 20 MgCl_2_, 10 NaHCO_3_, 5 trehalose, 115 sucrose, and 5 HEPES, pH adjusted to 7.2) by opening the third instar larvae dorsally along the midline and removing the entrails. Dissections were fixated with 4% paraformaldehyde in PBS (pH 7.2) for 10 min or 100% MeOH for 5 min for NMJ staining. After fixation, filets were washed with PBS plus 0.05% Triton X-100 (PBT) and blocked for 60 min in 5% normal goat serum (S2007; Sigma-Aldrich). For immunostainings, the larvae were incubated with primary antibodies at 4°C overnight and subsequently washed in a 0.05% PBT solution for 2 h at RT. Larvae were then incubated for 2–3 h with secondary antibodies at RT. Washing procedures were repeated. Larvae were finally mounted in Mowiol 4–88 (0713; Carl Roth).

#### Fab fragment–based blocking and double labeling with Myo15^C1^ (rabbit) and Myo15^C2^ (rabbit)

Primary antibody labeling was performed as above, but labeling was performed sequentially (https://www.jacksonimmuno.com/secondary-antibody-resource/wp-content/uploads/Fab-Blocking.pdf, [Gimber et al., 2022]). In short, after fixation, washing, and blocking as above, larvae were incubated with the primary Myo15^C1^ antibody for 1 h and washed subsequently three times for 20 min in washing solution (as above). Incubation with excess Fab1 goat–anti-rabbit Al488 (1:50) overnight at 4°C in blocking solution. In this step, the monovalent Fab1 fragments cover and block the surface of immunoglobulins, allowing a subsequent labeling with primary antibodies from the same host species. Therefore, the first Fab1 fragment (1:50) is given in excess in comparison to the second Fab1 fragment (1:200). Subsequent washing three times for 1 h in washing solution, 1 h blocking, and incubation with Fab1 goat–anti-rabbit Al647 (1:200) for 2–3 h at RT. Subsequent washing three times for 20 min and mounting as above. To confirm blocking efficiency, we performed the same protocol, only eliminating the primary antibody. Primary and secondary antibodies used in these studies are shown in Table 4.

**Table 4.** Primary and secondary antibodies

Primary antibodies	Source	Identifier
Immunofluorescence imaging		
Acetylated tubulin (anti-mouse, 1:500)	Sigma-Aldrich	T6793
ATP5 (ATP5A, ATP-synthase) (anti-mouse, 1:500)	Abcam	14748
BRP (Nc82) (anti-mouse, 1:250)	Developmental Studies Hybridoma Bank	Nc82
BRP (last 200) (anti-rabbit, 1:500)	S.J. Sigrist laboratory, Freie Universität Berlin, Germany (Ullrich et al., 2015)	last200 (rb)
DAP160 (anti-rabbit, 1:500)	Gift from O. Shupliakov laboratory, Karolinska Institutet, Solna, Sweden (Winther et al., 2015)	N/A
DsRed (anti-rabbit, 1:500)	Clontech	632496
Fasciclin II (Fas2, anti-mouse, 1:50)	Developmental Studies Hybridoma Bank	1D4 anti-fasciclin II
Futsch (anti-mouse, 1:250)	Developmental Studies Hybridoma Bank	22C10
GFP (anti-mouse, 1:500)	Invitrogen	A11120
Fab1 Al488 (goat–anti-rabbit, 1:50)	Jackson ImmunoResearch	111–547-008
Fab1 Al647 (goat–anti-rabbit, 1:200)	Jackson ImmunoResearch	111–607-008
Liprin-α (anti-rabbit, 1:500)	S.J. Sigrist laboratory, Freie Universität Berlin, Germany (Depner et al., 2014)	C-term, 8111
Myo15^C1^ (anti-rabbit, 1:25)	This study	rb 774
Myo15^C2^ (anti-rabbit, 1:50)	This study	rb 15073
Neuroglian (Nrg, anti-mouse, 1:50)	Developmental Studies Hybridoma Bank	BP 104
Rab3 (anti-rabbit, 1:500)	S.J. Sigrist laboratory, Freie Universität Berlin, Germany (Turrel et al., 2024)	N/A
Rbsn-5 (anti-rabbit, 1:1,000)	Gift from A. Nakamura laboratory, RIKEN Center for Developmental Biology, Kobe, Japan (Tanaka and Nakamura, 2008)	N/A
RIM-BP (anti-guinea pig, 1:500)	S.J. Sigrist laboratory, Freie Universität Berlin, Germany (Bohme et al., 2016)	RBP gp
VGlut (anti-rabbit, 1:500)	Gift from H. Aberle laboratory, Heinrich-Heine-Universität, Düsseldorf, Germany (Mahr and Aberle, 2006)	C-term, VGlut
Western blots		
ATP5A (ATP-synthase) (anti-mouse, 1:1000)	Abcam	14748
GFP (anti-mouse, 1:4,000)	Sigma-Aldrich	SAB4301795
α-tubulin (anti-mouse, 1:100,000)	Sigma-Aldrich	T5168
Secondary antibodies		
Immunofluorescence imaging		
Alexa 488 (anti-rabbit, 1:500)	Invitrogen	A-11008
Alexa 488 (anti-mouse, 1:500)	Invitrogen	A-11001
Alexa 488 (anti-guinea pig 1:500)	Invitrogen	A-11073
Alexa 594 (anti-rabbit, 1:500 for STED)	Invitrogen	A32754
Alexa 594 (anti-mouse, 1:500 for STED)	Invitrogen	A32742
Atto 647N (anti-rabbit, 1:500 for STED)	Sigma-Aldrich	40839
Atto 647N (anti-mouse, 1:500 for STED)	Sigma-Aldrich	50185
Cy3 (anti-rabbit, 1:500)	Abcam	ab6939
Cy3 (anti-mouse, 1:500)	Abcam	ab97035
antiGFP StarRED (1:500 for STED)	Abberior	STRED
HRP-Alexa647 (conjugated antibody), (1:500)	Jackson ImmunoResearch	123–605-021
Western blots		
HRP-coupled goat anti-mouse (1:5,000)	Jackson ImmunoResearch	#115-035-003
HRP-coupled goat anti-rabbit (1:5,000)	Jackson ImmunoResearch	#111-035–144

VGlut, vesicular glutamate transporter 1.

#### PhTx treatment for PHP

Larval dissections and immunostaining were performed as described previously (Bohme et al., 2019; Ramesh et al., 2023). Homeostatic plasticity was induced through pharmacological challenge with 50 μM PhTx (Philanthotoxin 433 TFA salt, Aobious, CAS no. 276684-27-6) in Ca^2+^-free HL3 at RT. Controls were similarly treated by substituting PhTx with dH_2_0. Briefly, the larvae were immobilized with insect pins on a rubber dissection pad, cut open dorsally between the dorsal tracheal trunks, avoiding excessive stretching or tissue damage. The semi-intact larvae were incubated with PhTx for 10 or 30 min. The preparation was completed by flattening the body wall using insect pins to expose the muscles. Fixation and further staining protocol as described above.

### Generation of Myo15-C1 and Myo15-C2 antibody

#### Anti-Myo15—C1 antibody (rabbit)

The Myo15-C1 antibody was generated by BioGenes Gesellschaft für Biopolymere mbH. Via peptide synthesis, the C-SGSEKEREKRERER-amide (5 mg) was produced, with a purity of at least 80%, quality control by HPLC and MS. This corresponds to aa 2107–2020, a sequence C-terminal to the split FERM-like domain. The peptide was subsequently conjugated to a carrier and used for the immunization of two rabbits. Antibodies were affinity purified (using CNBr-Sepharose columns), and a quality control by ELISA of the final antibody was performed.

#### Anti-Myo15—C2 antibody (rabbit)

Via DNA synthesis, the nucleotide sequence 9,099–9,435 bp (336 bp) of the RD isoform for the 112-aa long peptide (3034 aa −3145 aa of the PD isoform, directly after the FERM-domain of the Myo15 tail) was generated and cloned into the pET28a (N-terminal 6xHis-tag) vector using the EcoRI and NotI restriction sides by Eurofins Genomics. After expression in *Escherichia coli*, the peptide was used for immunization of two rabbits by BJ Diagnostik.

### Image acquisition and quantification

#### Image acquisition

Conventional confocal and STED images were acquired with Leica DMI 6000 (SP8) and TCS SP8 gSTED 3× microscopes (Leica Microsystems), respectively. For confocal scans, a HC PL APO CS2 63×/1.40-N.A. oil objective (Leica Microsystems) was used, for STED a HC PL APO CS2 100×/1.40-N.A. oil objective (Leica Microsystems). Images were acquired at ∼20°C, and fluorochromes are indicated in the antibody section described above. For signal detection, HYD (highly sensitive) 400–800-nm spectral descanned for green and red channels and PMT 400–800-nm spectral descanned for far red channels were used for confocal scans. For STED, HyD Sp GaAsP was used. The NMJ z-stacks had a step size of 0.2–0.3 μm between single optical slices. All images were acquired using the LAS X software (Leica Microsystems). The ImageJ 1.52p software was used for the analysis of confocal and STED images. GraphPad PRISM, version 8.42, was used for statistical analyses. For STED microscopy, Huygens Deconvolution software was used, applying a theoretical point spread function automatically computed based on pulsed- or continuous-wave STED-optimized function and the specific microscope parameters. The default deconvolution settings were applied. Images for figures were processed, if necessary, with ImageJ software to enhance brightness using the brightness/contrast function. Confocal stacks were processed with the image software Fiji (https://fiji.sc) (Schindelin et al., 2012). Image analysis followed the standard protocol as described by Andlauer and Sigrist (2012) and will be described in detail for the different analyses below.

#### NMJ image quantifications

Type1b NMJs on muscle 4 were analyzed. The HRP-Cy5 antibody signal was used as the template for a mask, restricting the quantified area to the shape of the NMJ. The original confocal stacks were converted to maximal projections, and after background subtraction (if required), a mask of the synaptic area/brain aggregates was created by applying a manual threshold to remove the irrelevant lower intensity pixels. For proteins with a muscle contribution (Rbsn-5, mitochondria, and Myo15 antibodies) in each slice of the Z-stack, an HRP mask outlining the NMJ in focus in this slice was created, and all signal outside the mask was deleted. These “cleaned” single slides were used for a maximum projection, now only including the signal from the NMJ within the HRP mask and not the underlying muscle tissue. The background signal for subtraction was determined in a maximum projection from the raw Z-stack.

The segmentation of single spots was done semiautomatically via the command “Find Maxima” embedded in the Fiji software and by hand with the pencil tool and a line thickness of 1 pixel. The processed picture was then transformed into a binary mask using the same lower threshold value as in the first step. This binary mask was then projected onto the original unmodified image using the “min” operation from the ImageJ image calculator. AZ area is the mean area value of all determined individual AZ areas of one NMJ. For EE analysis with Rbsn-5, a cut-off of 0.0202 µm^2^ was used. AZ density is the total number of AZs normalized to the NMJ (HRP) area of one NMJ. For BRP mean pixel intensity of single AZ, the mean pixel intensity of the individual AZs was determined and the mean value plotted. For NMJ length, lines were drawn along each branch of the NMJ, their length measured (using ImageJ), and added. NMJ area was determined as the area of the HRP mask. For the total number of AZs, all identified AZs of one NMJ were summed up. For total BRP intensity, the BRP mean pixel intensity within the HRP mask was calculated as “integrated density” using ImageJ as the integral of all BRP intensities within the HRP mask.

Mean pixel intensity of RFP signal upon Myo15 OE and simultaneous RNAi-mediated knock-down was determined as above described, values were normalized to Myo15^RFP^ overexpression, and the mean value of mean pixel intensity of the control (ok6-Gal4 × w1118) was subtracted from all values, as this value represents the unspecific background intensity.

For Myo15C1/C2 co-labeling with specific Fab1-tagged fragments and PhTx treatment, each antibody mean pixel intensity was normalized to its own signal or the corresponding -PhTx control.

Actin mean pixel intensity is the mean pixel intensity within the HRP mask (see above); total actin intensity within the HRP mask was calculated as integrated density using ImageJ as the integral of all actin intensities within the HRP mask. Single actin complex area was determined as single BRP-marked AZs (see above), and actin density is the total number of actin complexes normalized to the NMJ (HRP) area of one NMJ. If applicable, mean intensities were normalized to the HRP mean pixel intensity (of all intensity data within the analysis), but only if the HRP mean pixel intensity was not significantly altered between the compared groups.

#### Salivary gland image quantification

Salivary gland imaging as for NMJs. The original confocal stacks were converted to maximal projections. No background subtraction was performed, as the whole image was covered by the tissue, and no unambiguous background signal could be determined. Mean pixel intensity of maximum projection for the corresponding channel was determined as above. Data were not normalized and represent raw mean pixel intensity.

#### Pearson’s correlation coefficient

First steps as described by Andlauer and Sigrist (2012). In brief, maximum projections of NMJs for both channels were created (ImageJ). Images were background subtracted, and the mean pixel intensity of all images was normalized to the maximum value of 256. All signals outside of the HRP mask were deleted. Using ImageJ, co-localization threshold plugin, Myo15 signal was set to channel 1, other protein (e.g., actin) to channel 2, and Rcoloc (Pearson’s above threshold) was plotted. As control “inverted” the second channel was flipped horizontally and compared with the corresponding HRP masked ROI of the first channel. N represents NMJs with 1–2 NMJs/animal and 4–6 animals/genotype.

#### K-neighbor analysis

K-neighbor analysis was performed as previously described (Pooryasin et al., 2021). The spatial relation between Myo15 dots and the BRP (Nc82) C-terminal ring center was determined on 8-bit deconvolved STED images (see STED imaging above). Briefly, from an image with 1–2 boutons/image and three images/NMJ, 2 NMJs/animal, three animals in total, individual planar AZs were selected within 1.06 × 1.06-μm ROIs (53 × 53 pixels) in ImageJ (version 1.52p; NIH). Images were binned into three groups, small AZs (area = 1–159 pixel^2^, total 227 AZs), middle AZs (160–215 pixel^2^, total 301 AZs), and big AZs (<215 pixel^2^, total 305 AZs). To identify the exact position of the AZ center, even smaller AZ ROIs were placed on the BRP subimages, tightly surrounding the BRPNC82 rings, the lowest intensity pixel value subtracted from the image and the center of mass determined using output values XM and YM of the ImageJ function “Measure.” The same ROIs (53 × 53 pixels) were used to retrieve the “X” and “Y” coordinates of local intensity maxima in the second channel via the function Find maxima. Subsequently, the Euclidean distances of each AZ protein spot relative to the BRP ring center of mass, indicating the AZ center, were calculated using MATLAB (R2016a; MathWorks). For each AZ, the observed distances were ranked, and the minimum distance was selected. Values were averaged per animal.

#### Radial intensity profiles

Image analysis was performed with a custom ImageJ script, available at GitHub: Gimber, N. Cluster Detection and Radial Profiler. GitHub. https://github.com/ngimber/ClusterProfile. Myosin clusters were identified by first removing the background signal via auto-thresholding (“Moments” algorithm [Tsai, 1985]) and then using the Find Maxima function of ImageJ (Schneider et al., 2012) to determine cluster centers. Radial intensity profiles were measured in both channels (myosin and MTs) around the centers of myosin clusters. Median radial intensity profiles were calculated per image. Means and 95% confidence intervals from 25/28 (wild-type/*Myo15*^*ΔMyTH4* (*1+2*)^ mutant) images are plotted.

#### Statistical data analysis

All data were normalized to wild type (100%). N represents NMJs with 1–2 NMJs/animal and 5–6 animals/genotype. All graphs were plotted and statistically analyzed using GraphPad Prism version 8.4.2 for Windows. Data are presented as mean ± SEM. Normality was tested with the D’Agostino and Pearson omnibus normality test. If data were normally distributed and two groups were compared, an unpaired, two-tailed *t* test was used for statistical analysis (Fig. 1, C, E–I, K, M, N, P, Q, S, and U; Fig. S1, B, E–G, I, and K–M; Fig. 2, B, D–G, I, K, and M–P; Fig. S2, B, D, F, H, and K–N; Fig. 3, B–G, I, and K, Fig. 4, B and E; Fig. S3, C, F, H, J, N, and R; Fig. 5, B, D, F, I, and J; Fig. 6, B, and D–H; Fig. S4, A, C, E–I, K, M–Q, and S–V; Fig. 7 B; and Fig. S5 b). If data were normally distributed and more than two groups were compared, a one-way ANOVA with Turkey’s post hoc test was used (Fig. S3, T and U; Fig. 7, L and M; and Fig. S5, g–m). If data were not normally distributed and two groups were compared, a Mann–Whitney U test was used (Fig. 1 O; Fig. S4 P; Fig. S3 P; Fig. 5 H; Fig. 7, D–F and H–J; and Fig. S5, A–E, G, I–L, N, P–S, U, W–Z, and b). Significant outliers were determined in GraphPad Prism Outlier calculator (https://www.graphpad.com/quickcalcs/grubbs1/) with a significance level of alpha = 0.05. Significance is noted by *P < 0.05; **P < 0.01; ***P < 0.001; ****P < 0.0001.

### Domain prediction of Myo15 protein

For Myo15 domain prediction, we used the sequence analysis tool simple modular architecture research tool (https://smart.embl-heidelberg.de) (Letunic and Bork, 2018, Letunic et al., 2021), using the *Drosophila* PD-isoform of Myo15 and Hs MyoXVa isoform 1. This prediction tool identified the motor domain, IQ motifs, MyTH4 (1) domain, CC-domain, Src homology 3 domain, and the C-terminal MyTH4 (2)/FERM tandem domain. According to published sequence blast analysis and predictions by Liang et al. (1999), Planelles-Herrero et al. (2016) for human and (Rich et al., 2021) for *Drosophila* Myo15, describing an additional split FERM-like domain composed of partial F1 lobe adjacent to the first MyTH4 (1) domain and a distant partial F1 and F2/F3 lobe, we added this domain to our schematic representation.

### Western blotting

Six adult fly brains per genotype were dissected in cold hemolymph-like saline solution (HL3), collected in Low-bind Eppis (Sarstedt) and homogenized in lysis buffer (1x PBS, 0.5% Triton X-100, #T8787; Sigma-Aldrich, 2% SDS, 1x Protease inhibitor cocktail [#11836170001; Roche], and 1x sample buffer) for 1 h at 4°C on a rotator, followed by 5 min full-speed centrifugation at 18°C. Samples were stored at −20°C until further use. The volume equal to three adult brains was loaded onto precast 7.5% gradient gels (#4561023; Bio-Rad) and SDS-PAGE was performed at 80V/110V with 1x SDS running buffer (1 g SDS, 14,4 g glycine, 3,03 g Tris-Base Pufferan auf 1 liter H_2_O). Subsequently, transfer was performed as wet-transfer for 90 min at 300 mA and at 4°C with nitrocellulose membranes. Transfer buffer containing 20% methanol (14,4 g glycine, 3,03 g Tris-Base Pufferan, 200 ml methanol, and 800 ml H_2_O). For immunoblotting, membranes were blocked for 1 h in 5% milk solution (diluted in 0.1% PBS-Tween20, #8.22184; Sigma-Aldrich) and incubated with primary antibodies diluted in 5% milk solution overnight at 4°C. Membranes were washed six times for 5 min with 0.1% PBS-Tween20 and incubated with corresponding HRP-coupled secondary antibodies diluted in 5% milk solution for 1 h at RT. Membranes were washed again six times for 5 min with 0.1% PBS-Tween20, and blots were subsequently developed using ECL solution (#32132; Thermo Fisher Scientific) and Kodak/GE films.

### Electrophysiology

TEVC as well as single electrode (“current-clamp”) recordings were performed at RT on muscle 6 of third instar larval NMJs in the abdominal segments A2 and A3. Third instar larvae were dissected in modified Ca^2+^-free hemolymph-like saline (HL3; in mM: NaCl 70, KCl 5, NaHCO_3_ 10, MgCl_2_ 20 [TEVC] or 10 [current clamp], sucrose 115, trehalose 5, and HEPES 5). For PhTx experiments, larvae were cut open along the midline and incubated for the indicated time (10 or 30 min) in HL3 containing either 50 µM PhTx or the equivalent volume of water for controls. The incubation solution was gently perfused into the preparation using a pipette at the start of the incubation and once more at the half-time point (after 5 or 15 min). The preparation was then finished during the last 2 min of incubation time and afterward washed three times with HL3 before being transferred to bath solution for electrophysiological recordings. Recordings were obtained with a bath solution of HL3 with 1.5 (TEVC) or 0.4 (current clamp) mM CaCl_2_. Recordings were made from cells with an initial Vm between −50 (TEVC) or −40 (current clamp) and −80 mV, and input resistances of ≥4 MΩ, using intracellular electrodes with resistances of 30–50 MΩ, filled with 3 M KCl. Two cells were recorded per animal. Glass electrodes were pulled using a Flaming Brown Model P-97 micropipette puller (Sutter Instrument). Recordings were made using an Axoclamp 2 B amplifier with HS- 2A ×0.1 head stage (Molecular Devices) on a BX51WI Olympus microscope with a 40X LUMPlanFL/IR water immersion objective (Olympus Corporation). mEJCs/mEPSPs were recorded for 90 s (for TEVC, the voltage was clamped at −80 mV for mEJCs and at −60 mV for single, paired, and train eEJCs). eEJCs/eEPSPs were recorded after stimulating the appropriate motor neuron bundle with 5 (eEJCs) or 8 (eEPSPs) V, 300 µs at 0.2 Hz using an S48 Stimulator (Grass Instruments, Astro-Med, Inc.). To estimate the RRP size, single train recordings with 61 stimulations were performed at 100 Hz. Signals were digitized at 10 kHz using an Axon Digidata 1322 A digitizer (Molecular Devices) and low-pass filtered at 1 kHz using an LPBF-48DG output filter (NPI Electronic). The recordings were analyzed with pClamp 10 (Molecular Devices), GraphPad Prism 6 (GraphPad Software, Inc.), and two Python scripts utilizing the pyABF package for Python 3.10 (Harden, SW (2022, https://swharden.com/pyabf/#how-to-cite-pyabf)). pyABF 2.3.5. (available: https://pypi.org/project/pyabf). Stimulation artifacts of eEJCs/eEPSPs were removed for clarity. mEJCs/mEPSPs were further filtered with a 500-Hz Gaussian low-pass filter. Using a single template for all cells, mEJCs/mEPSPs were identified and averaged, generating a mean mEJC/mEPSP trace for each cell. An average trace was generated from 20 eEJC/eEPSP traces per cell for 0.2-Hz stimulation and 10-ms ISI paired pulse recordings and from 10 traces for 30-ms ISI paired pulse recordings. Rise time was calculated from the average trace of the 0.2-Hz stimulation recording as the time from 10 to 90% of the total amplitude before the peak. Decay constant τ was calculated by fitting a first-order decay function to the region of the average trace of the 0.2-Hz stimulation recording from 60 to 5% of the total amplitude after the peak. The amplitude of the average eEJC/eEPSP trace from the 0.2 Hz stimulation recording was divided by the amplitude of the averaged mEJC/mEPSP for each respective cell to determine the quantal content. 10- and 30-ms ISI paired pulse ratios were calculated by dividing the amplitude after the second pulse by the amplitude after the first pulse. The baseline for the second amplitude was set at the last point before the second stimulation artifact onset. Estimation of the RRP size and refilling rates was performed as described previously (Matkovic et al., 2013); briefly, the amplitudes of the responses to each stimulation in the train were extracted from the recording by using the last data points before each artifact onset as a baseline. For each cell, these amplitudes were then added cumulatively and divided by the average mEJC amplitudes as measured for this cell to obtain the cumulative quantal content. This cumulative quantal content was then plotted against the number of stimulations, and a linear regression was performed for the last 20 amplitudes, where the cells reach a steady state due to the depletion of the RRP. The intersect of this linear fit with the Y axis is the estimated RRP size, and the slope of the linear fit is the estimated refilling rate.

### Olfactory associative aversive conditioning

The *tubulin-Gal80*^*ts*^*;OK107* driver (*Gal80*^*ts*^*;OK107*) was used for conditional expression in the MB. To induce RNAi expression specifically in adults, the TARGET system was used as described by McGuire et al. (2003): flies were kept for 9 days at 29°C before conditioning for all RNAi-expressing flies. Flies were trained using the classical olfactory aversive conditioning protocols described previously (Tully and Quinn, 1985). Training and testing were performed in climate-controlled boxes at 25°C in 80% humidity under dim red light. At 2–3 days old, flies were transferred to fresh food vials and either kept at 29°C for RNAi induction or kept at 18°C for the non-induced controls. Conditioning was performed on groups of around 40–50 flies with 3-octanol (around 95% purity; Sigma-Aldrich) and 4-methylcyclohexanol (99% purity; Sigma-Aldrich). Odors were diluted at 1:100 in paraffin oil and presented in 14-mm cups. A current of 120 AC was used as a behavioral reinforcer. Memory conditioning and tests were performed with a T-maze apparatus (Tully and Quinn, 1985). In a single-cycle training, groups of flies were presented with one odor (CS^+^) paired with an electrical shock (US; 12 times for 1 min). After 1 min of pure air-flow, the second odor (CS^−^) was presented without the shock for another minute. During the test phase, flies were given 1 min to choose between two arms, giving each a distinct odor. An index was calculated as the difference between the numbers of flies in each arm divided by the sum of flies in both arms. The average of two reciprocal experiments gave a performance index. The values of the performance index range from 0 to 1, where 0 means no learning (50:50 distribution of flies) and a value of 1 means complete learning (all flies avoided the conditioned odor). For STM, flies were tested directly after conditioning, whereas for MTM 1-h flies were transferred to small tubes without food for 1 h before testing. For olfactory acuity and shock reactivity, around 50 flies were put in a choice position between either one odor and air for one min or electric shocks and no shocks, respectively.

### Online supplemental material

Supplemental material includes supplemental figures S1–S5 and figure legends. Fig. S1 shows that the *Myo15*^−/−^ mutants show defects in NMJ morphology and AZ scaffold assembly, which are rescued by presynaptic restoration of Myo15 level. Fig. S2 shows the analysis of AZ, SV, endocytic and cell adhesion proteins, and mitochondria upon motoneuronal Myo15 knock-down. Fig. S3 shows the Myo15 localization at the NMJ and salivary gland and antibody verification. Fig. S4 shows the MyTH4 (1) and FERM domain deletion mutants and Myo15 localization to presynaptic MTs. Fig. S5 shows the electrophysiological TEVC analysis of Myo15 loss-of-function, PhTx treatment of *Myo15*^*−/−*^ mutant, and sustained memory consolidation for Myo15 knock-down.

## Supplementary Material

SourceData F4 is the source file for Fig. 4.

SourceData FS3 is the source file for Fig. S3.

## Data Availability

The data underlying figures, tables, and graphs are available in the published article and its online supplemental material. All confocal.lif files, statistical prism files, quantification excel sheets, western blot images, and the macro for radial analysis are available at GitHub: https://github.com/ngimber/ClusterProfile.
