# Supplementary material for: Myosin 15 participates in assembly and remodeling of the presynapse
Source: J Cell Biol. 2025 Jul 8;224(9):e202305059. doi: 10.1083/jcb.202305059 (PMC12237259; doi:10.1083/jcb.202305059)
Supplement: SourceData F4 — is the source file for Fig. 4. [file jcb_202305059_sourcedataf4.pdf]

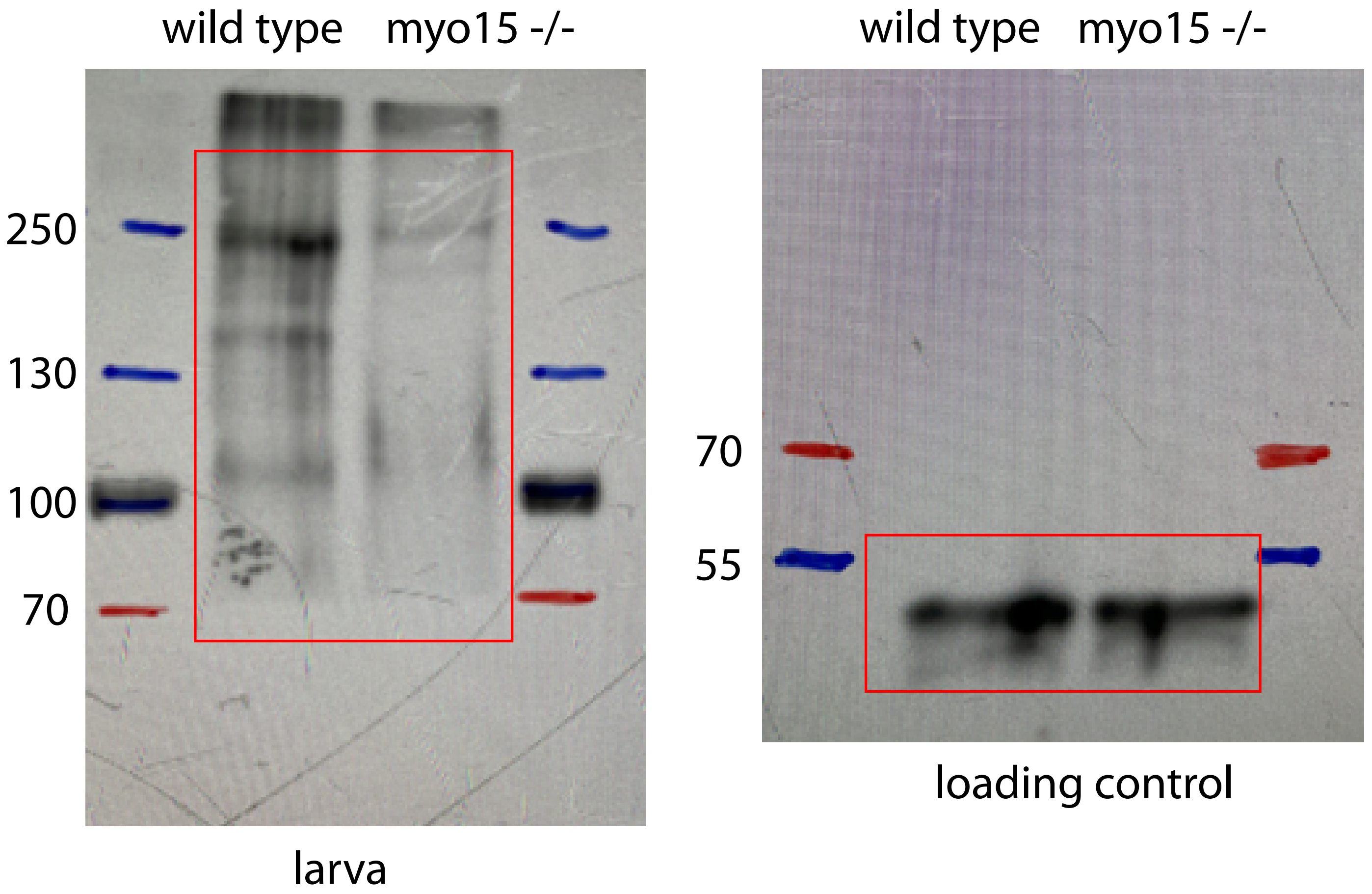

SourceDataF4C

control OE

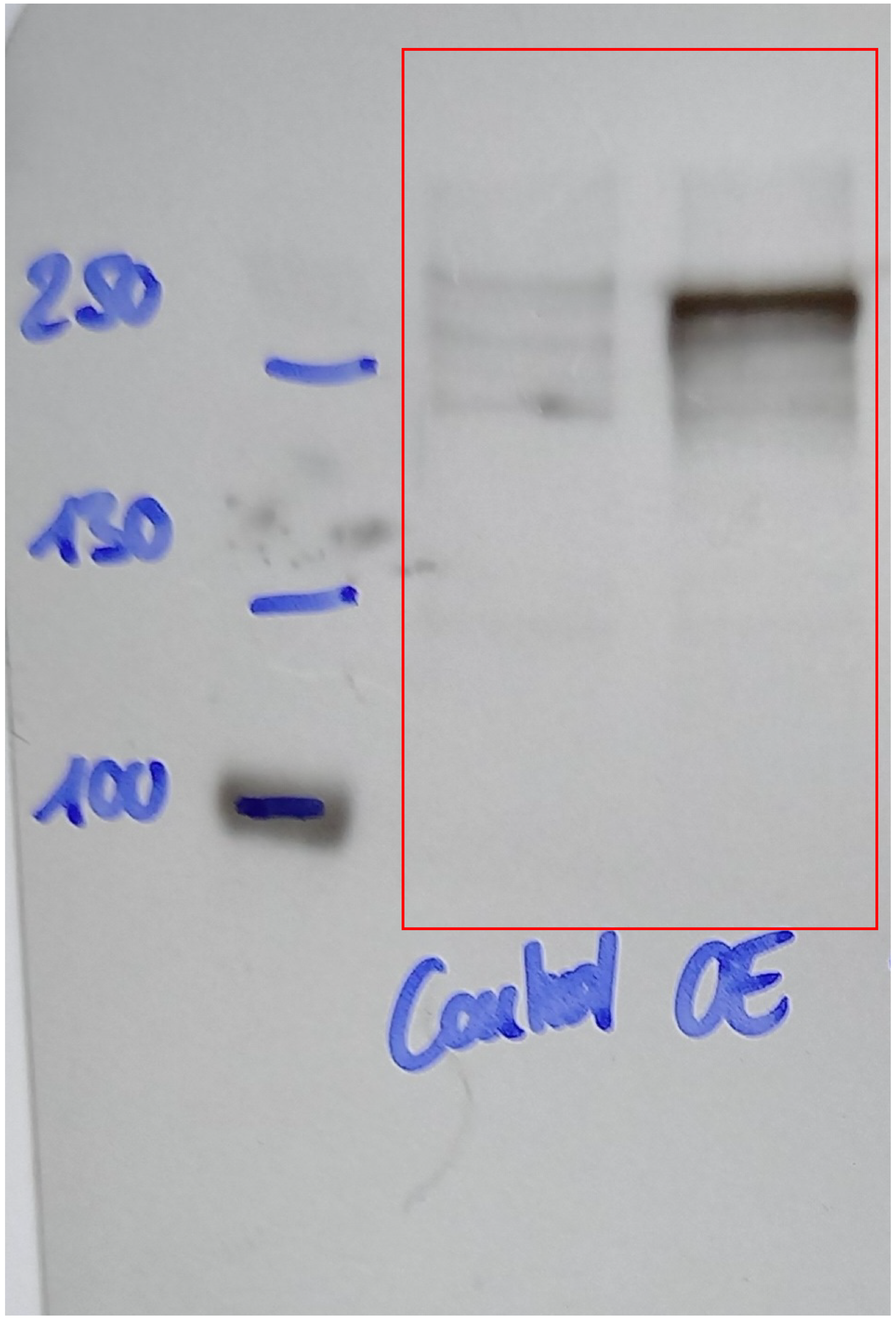

adult brains OE

control OE

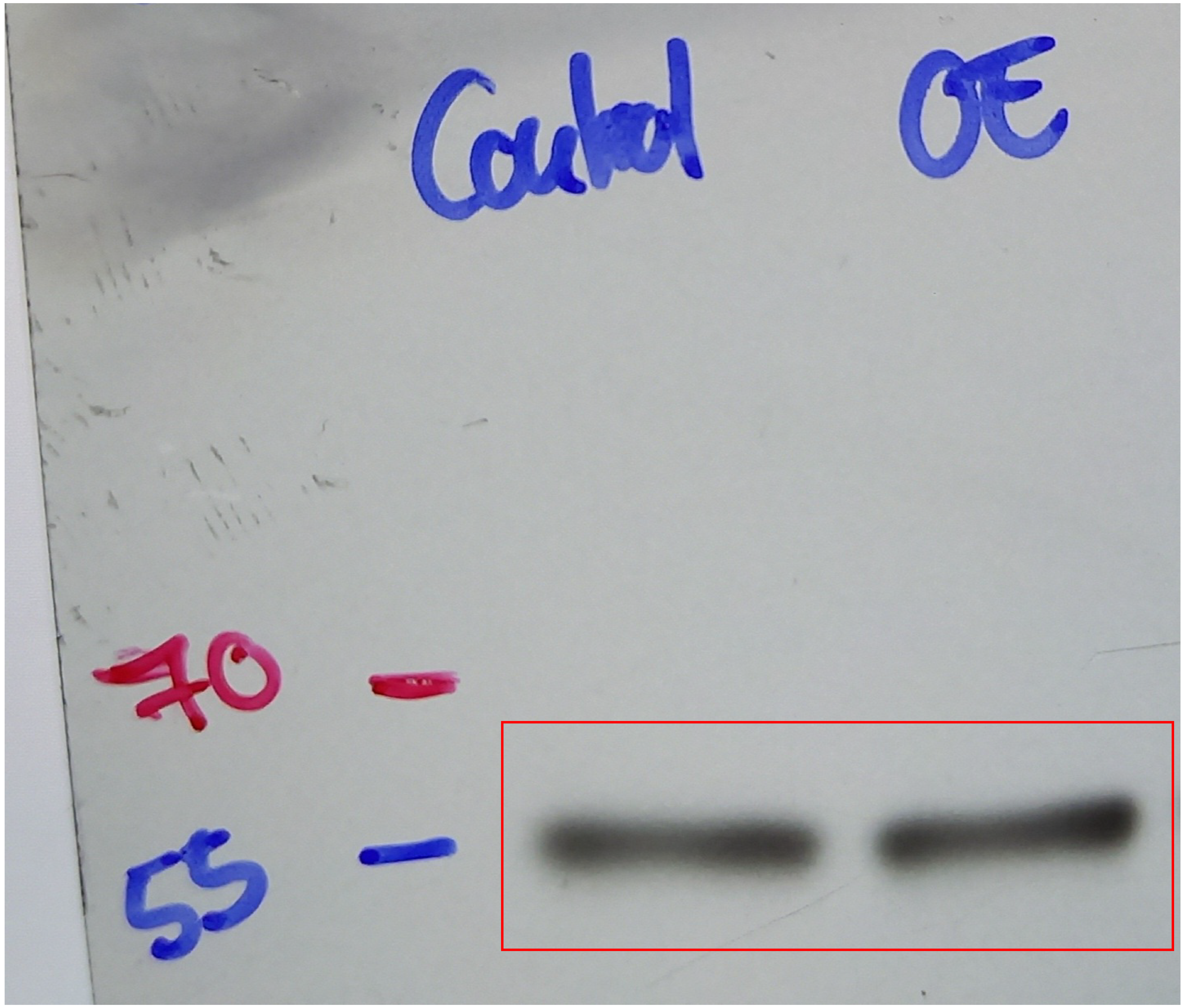

loading control

SourceDataF4F
