# Supplementary material for: Myosin 15 participates in assembly and remodeling of the presynapse
Source: J Cell Biol. 2025 Jul 8;224(9):e202305059. doi: 10.1083/jcb.202305059 (PMC12237259; doi:10.1083/jcb.202305059)
Supplement: SourceData FS3 — is the source file for Fig. S3. [file jcb_202305059_sourcedatafs3.pdf]

wild type myo15 -/-

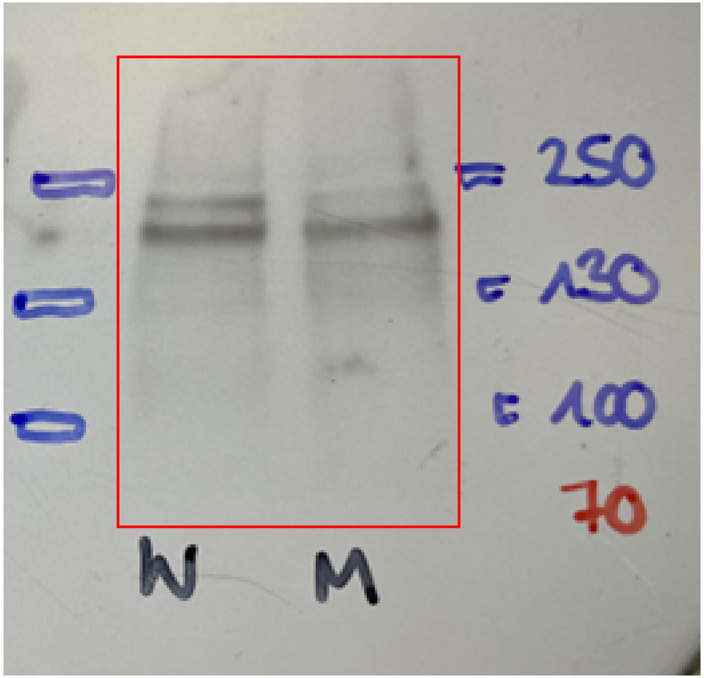

larva

wild type myo15 -/-

70
55

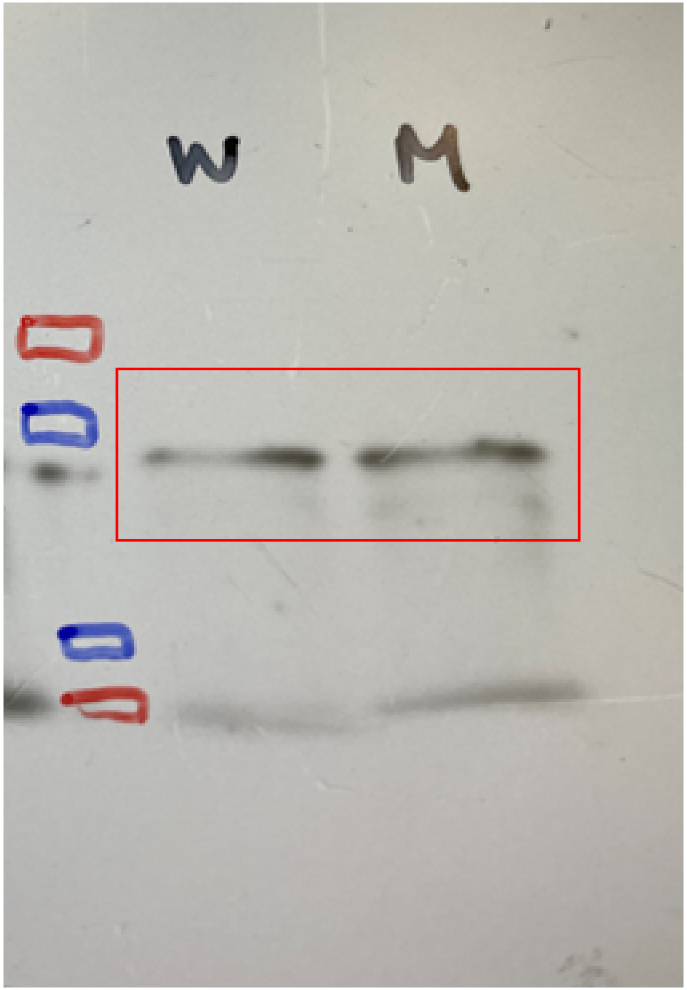

loading control

SourceDataFS3D

control OE

250
130
100
70

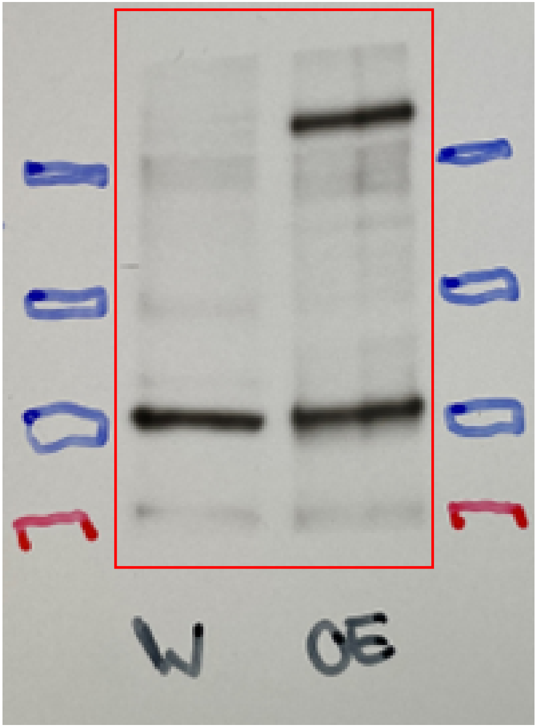

adult brains OE

control OE

70
55

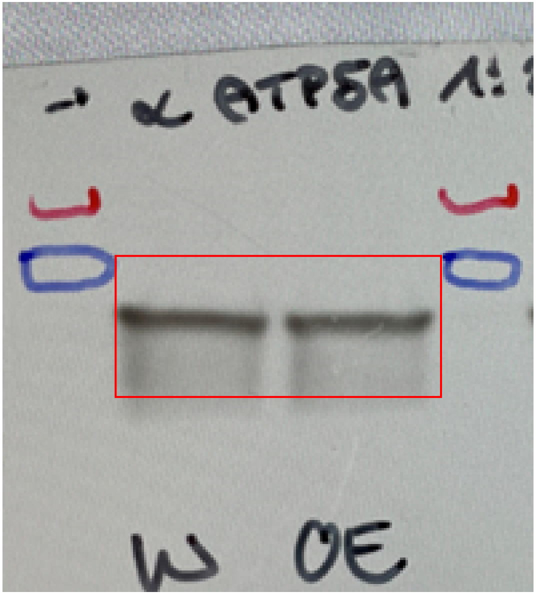

loading control

SourceDataFS3K
